# Intelligent bacteria‐targeting ZIF‐8 composite for fluorescence imaging‐guided photodynamic therapy of drug‐resistant superbug infections and burn wound healing

**DOI:** 10.1002/EXP.20230113

**Published:** 2024-04-19

**Authors:** Xiaoxue Li, Wei Wang, Qiuxia Gao, Shanshan Lai, Yan Liu, Sitong Zhou, Yan Yan, Jie Zhang, Huanhuan Wang, Jiamei Wang, Yi Feng, Ronghua Yang, Jianyu Su, Bin Li, Yuhui Liao

**Affiliations:** ^1^ Molecular Diagnosis and Treatment Center for Infectious Diseases Dermatology Hospital of Southern Medical University Guangzhou Guangdong China; ^2^ School of Inspection Ningxia Medical University Yinchuan Ningxia China; ^3^ Institute for Health Innovation and Technology National University of Singapore Singapore Singapore; ^4^ Department of Dermatology The First People's Hospital of Foshan Foshan Guangdong China; ^5^ Department of Burn and Plastic Surgery Guangzhou First People's Hospital South China University of Technology Guangzhou Guangdong China; ^6^ School of Food Science and Engineering South China University of Technology Guangzhou Guangdong China

**Keywords:** bacterial infection imaging, multifunctional therapeutic system, pH‐responsive, targeting bacteria, wound healing

## Abstract

Infected burn wounds are characterized by persistent drug‐resistant bacterial infection coupled with an inflammatory response, impeding the wound‐healing process. In this study, an intelligent nanoparticle system (CCM+TTD@ZIF‐8 NPs) was prepared using curcumin (CCM), an aggregation‐induced emission luminogens (TTD), and ZIF‐8 for infection‐induced wound healing. The CCM+TTD@ZIF‐8 NPs showed multiple functions, including bacteria targeting, fluorescence imaging and pH response‐guided photodynamic therapy (PDT), and anti‐inflammatory. The positive charges of ZIF‐8 NPs allowed the targeting of drug‐resistant bacteria in infected wounds, thereby realizing fluorescence imaging of bacteria by emitting red fluorescence at the infected site upon blue light irradiation. The pH‐responsive characteristics of the CCM+TTD@ZIF‐8 NPs also enabled controllable CCM release onto the infected wound site, thereby promoting the specific accumulation of ROS at the infected site, with outstanding bactericidal efficacy against drug‐resistant *Staphylococcus aureus (S. aureus)* and *Pseudomonas aeruginosa (P. aeruginosa)* strains in vitro/in vivo. Additionally, due to the excellent bactericidal effect and anti‐inflammatory properties of CCM+TTD@ZIF‐8 NPs combined with blue light irradiation, the regeneration of epidermal tissue, angiogenesis, and collagen deposition was achieved, accelerating the healing process of infected burn wounds. Therefore, this CCM+TTD@ZIF‐8 NPs with multifunctional properties provides great potential for infected burn wound healing.

## Graphical abstract

An intelligent acid responsive ZIF‐8 nanoparticle (CCM + TTD@ZIF‐8 NPs) is proposed and is used to promote the healing of burns and infected wounds. It can effectively target bacteria and achieve bacterial imaging under blue light irradiation, effectively releasing CCM and TTD in acidic environments, thereby inhibiting the inflammatory microenvironment and promoting wound healing.

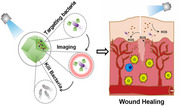

## 1 INTRODUCTION

Following severe burns, the skin loses the ability to resist the invasion of foreign pathogens and provides a humid environment for the settlement of bacteria, which could lead to the occurrence of bacterial infections and subsequent inflammatory responses. In the early stages of wound repair, M1‐type macrophage plays a major role in controlling bacterial infection, but its ability to phagocytose bacteria is greatly reduced after persistent pathogen invasion, thereby promoting the formation of biofilm on the envelope surface, which seriously delays the wound repair process.^[^ ^1^ ^]^ Traditional antibiotics have made remarkable achievements in the fight against pathogen infections but are confronted with the emergence of drug resistance due to the abuse of antibiotics, a major challenge for global healthcare systems.^[^ ^2^ ^]^ Therefore, it is of great significance to develop treatment strategies that efficiently kill drug‐resistant bacteria and regulate the microenvironment of burn inflammation.

Photodynamic therapy (PDT) can induce DNA damage, lipid peroxidation, and protein dysfunction in bacterial cells through the production of reactive oxygen species (ROS), which could avoid the emergence of resistant strains.^[^ ^3^ ^]^ As a fluorescence polyphenolic compound, curcumin (CCM) has been proven to be a natural photosensitizer that exert excellent antimicrobial PDT under blue light irradiation.^[^ ^4^ ^]^ Meanwhile, CCM has an anti‐inflammatory function, increases vascular density and fibroblast proliferation, and accelerates skin regeneration.^[^ ^5^ ^]^ Nevertheless, individual application of CCM has several challenges, such as the strong toxicity and weak biodegradation of CCM, that hamper clinical application.^[^ ^6^ ^]^ Additionally, the low hydrophilicity and weak photostability of CCM result in the limited production of ROS,^[^ ^7^ ^]^ and the non‐specific accumulation and short lifetime of ROS decrease antibacterial efficacy and impair normal human tissues.^[^ ^8^ ^]^ Another challenge of PDT is the lack of imaging‐guided function in the infected site during the antibacterial therapy process. The fluorescent detection of bacteria can efficiently find lesion sites and achieve real‐time diagnosis. In contrast to traditional fluorogens, fluorogens with aggregation‐induced emission (AIEgens) can emit bright fluorescence upon aggregation formation. Due to the unique AIE property combined with excellent photostability and biocompatibility, AIEgens have been successfully applied for cell tracing and imaging‐guided therapy.^[^ ^9^ ^]^ Thus, it is necessary to find a suitable carrier for the loading of photosensitizers to improve photostability and target bacteria to achieve efficient PDT.

Recently, numerous types of smart carrier materials have been reported, including materials with high responses to magnetic fields,^[^ ^10^ ^]^ light,^[^ ^11^ ^]^ pH,^[^ ^4^, ^12^ ^]^ or temperature,^[^ ^13^ ^]^ thus circumventing unintended side effects of antimicrobial treatment. Due to the acidic state (pH 5.1) of infected wounds^[^ ^14^ ^]^ pH‐responsive drug carriers can be used to maximize drug release within the wound area. Zeolitic imidazolate framework 8 (ZIF‐8), formed by the coordination interaction of zinc ions (Zn^2+^) and 2‐methylimidazole,^[^ ^4^ ^]^ is a metal‐organic framework material (MOF) and a pH‐responsive porous nanocarrier. It possesses high chemical stability and a unique porous structure, with promising features as a carrier for efficient PDT in infected wounds by reducing the self‐quenching and self‐aggregation of photosensitizers and increasing their solubility and photostability.^[^ ^15^ ^]^ The pore size and aperture of MOFs are significantly smaller, which can be accurately controlled ^[^ ^16^ ^]^ compared to polymeric structures,^[^ ^17^ ^]^ making the drug loading performance of MOFs superior.^[^ ^18^ ^]^ Additionally, the positively charged state of ZIF‐8 allows the targeting of negatively charged bacteria, thus directing the delivery of photosensitizer toward pathogens in infected sites. It has been reported in the literature that the pH of the wound changes due to the infected state of the wound. During the hemostatic and inflammatory phases, the wound microenvironment is acidic due to increased glycolysis and lactic acid accumulation as a result of vasoconstriction and inadequate blood supply. During the proliferative and remodeling phases, the pH gradually increases and becomes alkaline due to vasodilation, blood vessel formation, acidic pus clearance, and reactivation of aerobic metabolism, thus utilizing this property to make ZIF‐8 pH‐sensitive, which can “turn on” the release of photosensitizers and Zn^2+^ in acidic environments, achieving specific accumulation of ROS at the infected site. Besides, Zn^2+^ is involved in cell growth and differentiation, nerve regeneration, and broad‐spectrum antimicrobial ability,^[^ ^19^ ^]^ making it suitable for the current application. Therefore, ZIF‐8 in this study possesses a great advantage as a smart carrier for the transport of photosensitizers.

**SCHEME 1:**
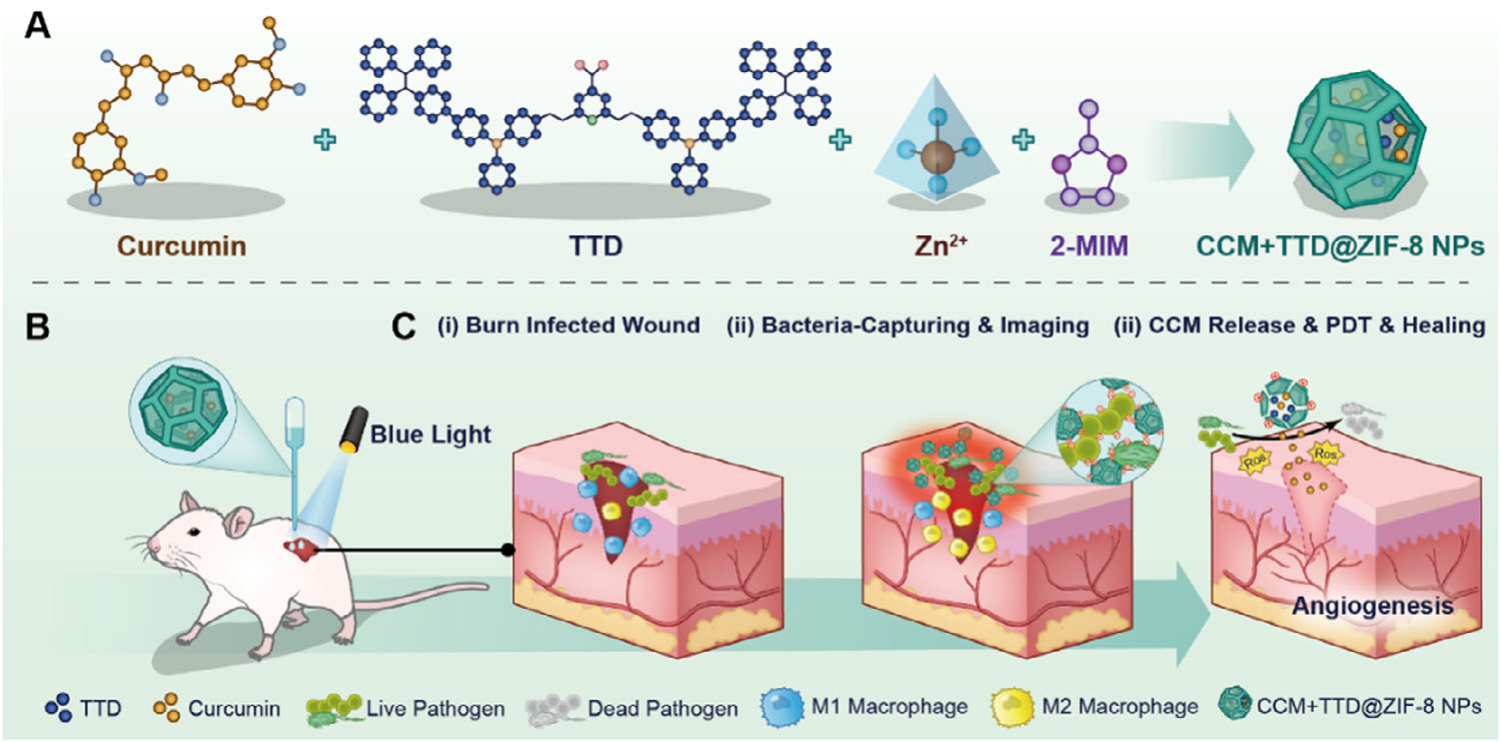
(A) Illustrations of the synthesis of CCM+TTD@ZIF‐8 NPs. (B) CCM+TTD@ZIF‐8 NPs combined with blue light for the repair of infected burn wounds. (C) Mechanism of CCM+TTD@ZIF‐8 NPs combined with blue light for the repair of infected burn wounds.

In this study, a kind of AIE fluorophore, 2‐(2,6‐bis((E)−4‐(phenyl(4′‐(1,2,2‐triphenylvinyl)‐[1,1′‐biphenyl]−4‐yl)amino)styryl)−4H‐pyran‐4 ylidene)malononitrile (TTD), was selected for the imaging of bacteria. Eventually, an intelligent nanoparticle system (CCM+TTD@ZIF‐8 NPs) was constructed using CCM, a kind of aggregation‐induced emission luminogens called TTD, and ZIF‐8. The CCM+TTD@ZIF‐8 NPs were shown to have multiple functions, including bacteria targeting, fluorescence imaging, and pH response‐guided PDT, and anti‐inflammation, to augment the healing of wounds infected with drug‐resistant bacteria (Scheme 1). This multifunctional nanoparticle has broad application prospects for treating burns and infected wounds.

## 2 RESULTS

### 2.1 Synthesis and characterization of CCM+TTD@ZIF‐8 NPs

**FIGURE 1:**
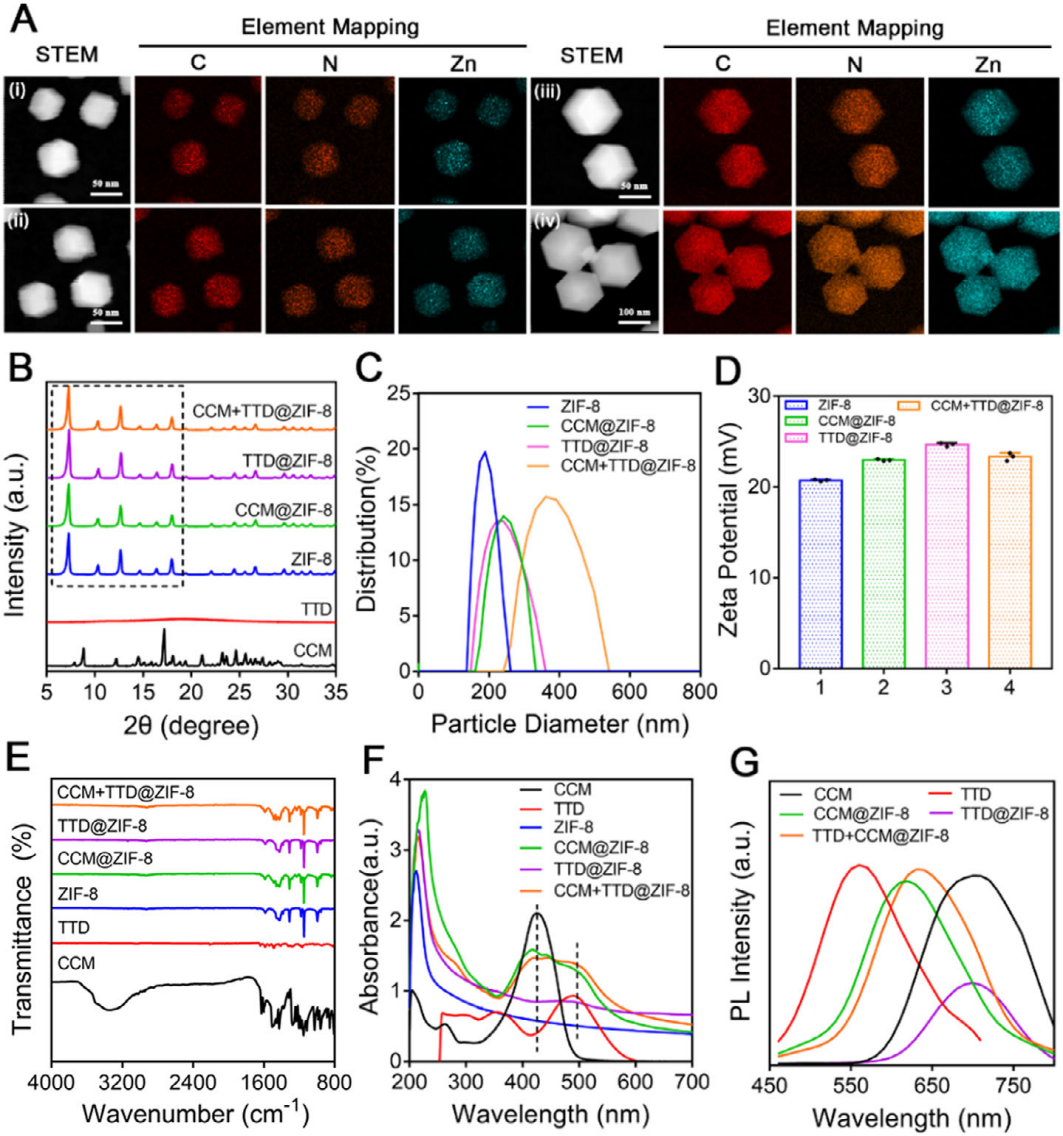
Characterization of nanoparticles. (A) STEM images and corresponding EDX elemental mapping of (i) ZIF‐8, (ii) TTD@ZIF‐8 NPs, (iii) CCM@ZIF‐8 NPs, and (iv) CCM+TTD@ZIF‐8 NPs. (B) XRD patterns. (C) Particle size distribution. (D) ζ potentials (*n* = 4). (E) FTIR spectra. (F) UV–vis absorption spectra. (G) Photoluminescence spectra. STEM: scanning transmission electron microscopy, EDX: energy dispersive X‐ray spectroscopy, ZIF‐8: Zeolitic imidazolate framework 8, TTD: 2‐(2,6‐bis((E)−4‐(phenyl(4′‐(1,2,2‐triphenylvinyl)‐[1,1′‐biphenyl]−4‐yl)amino)styryl)−4H‐pyran‐4 ylidene)malononitrile, CCM: curcumin, XRD: X‐ray diffraction, FTIR: fourier transform infrared spectrometer, UV‐vis: ultraviolet‐visible spectroscopy.

ZIF‐8 NPs were synthesized via a single‐step procedure and presented a regular dodecahedral crystal with a diameter of 50 nm (Figure 1A(i)). The unique porous structure and solubility of ZIF‐8 were used to prepare CCM+TTD@ZIF‐8 NPs with excellent PDT and imaging via a similar route (Scheme 1A).^[^ ^21^ ^]^ The interaction of Zn^2+^ with CCM and TTD led to the formation of the core, while the hydrophilic 2‐MIM was used as the skeleton to form the shell through interaction with Zn^2+^. The X‐ray diffraction (XRD) pattern (Figure 1B) was similar to that in the previous report,^[^ ^20^ ^]^ suggesting that ZIF‐8 NPs were successfully synthesized. XRD showed similar crystalline characteristic diffraction peaks in all ZIF‐8 NPs (CCM@ZIF‐8, TTD@ZIF‐8, and CCM+TTD@ZIF‐8 NPs; Figure 1B), indicating that the crystal structure of ZIF‐8 was not destroyed by CCM and TTD loading. The four kinds of NPs showed a dodecahedral crystal structure, with a larger particle size of CCM@ZIF‐8, TTD@ZIF‐8, and CCM+TTD@ZIF‐8 NPs compared to that of ZIF‐8 NPs. The hydrodynamic sizes of ZIF‐8 NPs, CCM@ZIF‐8 NPs, TTD@ZIF‐8 NPs, and CCM+TTD@ZIF‐8 NPs were ≈188.76, 240.68, 221.95 and 360.84 nm, respectively (Figure 1C). This increase in size may be due to the incorporation of CCM and TTD. It was found that the surface ζ potential of CCM@ZIF‐8 NPs, TTD@ZIF‐8 NPs and CCM+TTD@ZIF‐8 NPs was positive (Figure 1D), which indicated that the surface ζ potential of ZIF‐8 was not destroyed by CCM and TTD loading, and this result also implied that CCM+TTD@ZIF‐8 NPs may adsorb on bacteria surfaces with negative charges. The FTIR spectra of CCM@ZIF‐8 NPs and CCM+TTD@ZIF‐8 NPs (Figure 1E) exhibited an absorption peak at 1500 cm^−1^, not present in ZIF‐8 NPs, attributed to an aromatic hydrocarbon (CCM). However, the phenolic group peaks at 3600 cm^−1^ were not found in CCM@ZIF‐8 NPs and CCM+TTD@ZIF‐8 NPs, suggesting that the interaction of Zn^2+^ in ZIF‐8 and the phenolic group in CCM contributed to the incorporation of CCM (Figure 1E and Figure S1).

The optical properties of the composite NPs were then assessed. The characteristic absorption peak at 229 nm was observed for ZIF‐8, while CCM and TTD had a strong absorption peak at 425 and 488 nm, respectively (Figure S2). A red shift of the absorption peak was observed following the incorporation of CCM and TTD into ZIF‐8 NPs (Figure 1F). TTD and CCM performed robust fluorescence emission peaked at about 555 and 670 nm, respectively, following excitation at 425 nm.^[^ ^22^ ^]^ The photoluminescence spectrum of CCM@ZIF‐8 NPs and CCM+TTD@ZIF‐8 NPs were also blue‐shifted by 100 nm compared to that of CCM (Figure 1G). A previous study explained that the blue shift may be due to a decrease in the band gap between the p–p* electronic transition via the interaction of OH─ in CCM and Zn^2+^ in CCM.^[^ ^20^, ^23^ ^]^ These results indicated that CCM and TTD were successfully encapsulated into the ZIF‐8 NPs.

To calculate the drug‐loading capacity (DLC) and drug‐loading encapsulation (DLE) of CCM@ZIF‐8 NPs and CCM+TTD@ZIF‐8 NPs, the concentration‐absorbance standard curve of CCM at 425 nm was determined over the concentration range 0−10 µg mL^−1^ (Figure S3). According to the equation of the standard curve (*A* = 0.08991*c* + 0.009061, *R* ^2 ^= 0.998), the DLC and DLE of CCM@ZIF‐8 NPs were 4.77% and 19.07%, respectively, while the respective values for CCM+TTD@ZIF‐8 NPs were 5.12% and 20.46% (Table S1).

### 2.2 pH‐dependent release, singlet oxygen (^1^O_2_) production, and imaging of targeting bacteria

**FIGURE 2:**
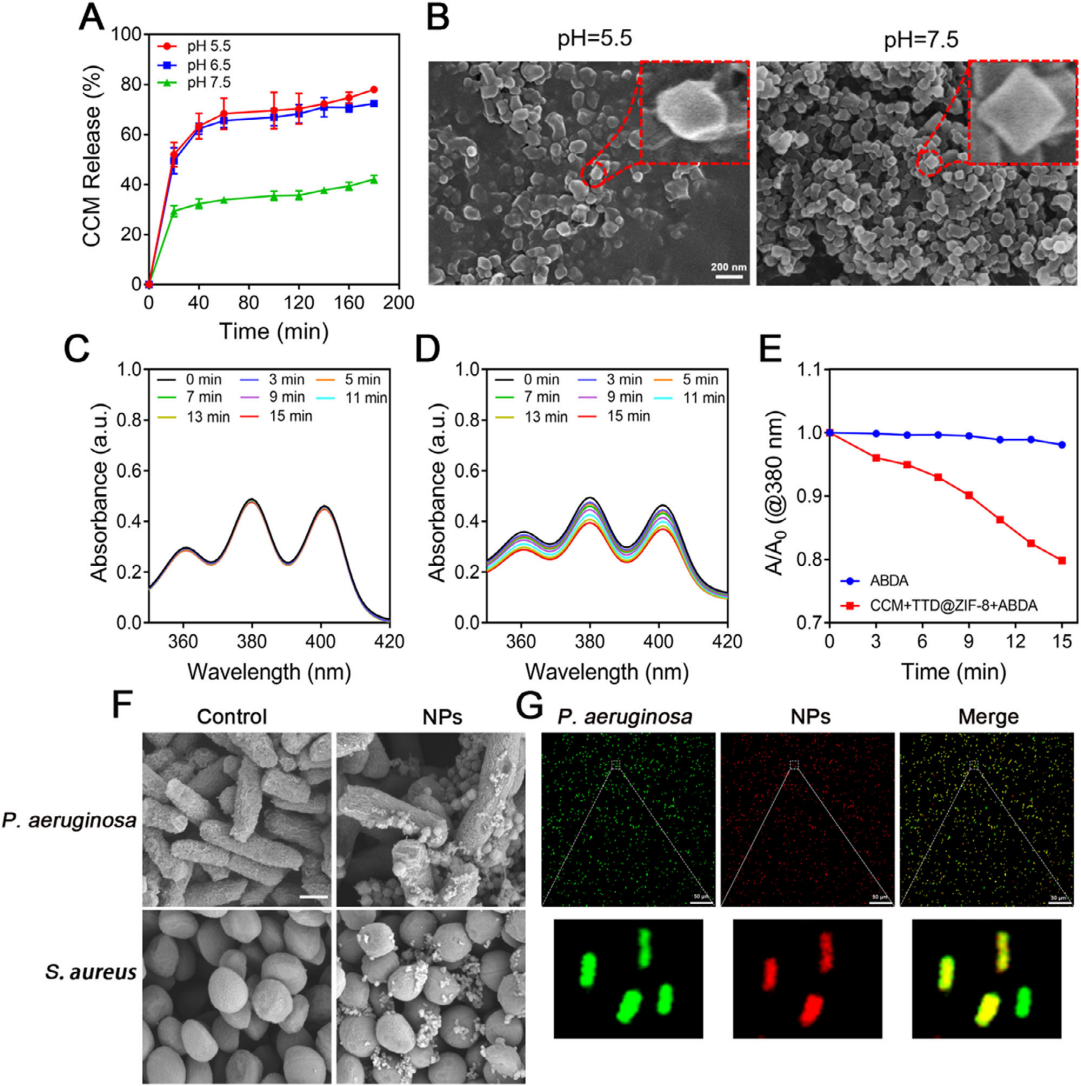
pH‐responsive release, photodynamic, bacteria‐capturing, and fluorescence imaging characteristics of CCM+TTD@ZIF‐8 NPs. (A) CCM release profile from CCM+TTD@ZIF‐8 NPs in PBS at pH 5.5, 6.5, and 7.5. (B) Morphology of CCM+TTD@ZIF‐8 NPs in PBS at pH 5.5 and 7.5. (C) UV–vis absorption spectra of ABDA under blue light irradiation (20 mW cm^−2^) with different times (0, 3, 5, 7, 9, 11, 13, and 15 min). (D) UV–vis spectrum of ABDA in the presence of CCM+TTD@ZIF‐8 NPs under blue light irradiation (20 mW cm^−2^) at different times (0, 3, 5, 7, 9, 11, 13, and 15 min). (E) Comparison of ROS generation ability between ABDA and ABDA+NPs under blue light irradiation. ABDA was solubilized with CCM+TTD@ZIF‐8 NPs in PBS at pH 7.4. (F) SEM images of *P. aeruginosa* and *S. aureus* following co‐incubation with CCM+TTD@ZIF‐8 NPs. (G) Fluorescence images of *P. aeruginosa* labeled with green fluorescence following co‐incubation with CCM+TTD@ZIF‐8 NPs. ZIF‐8: Zeolitic imidazolate framework 8, TTD: 2‐(2,6‐bis((E)−4‐(phenyl(4′‐(1,2,2‐triphenylvinyl)‐[1,1′‐biphenyl]−4‐yl)amino)styryl)−4H‐pyran‐4 ylidene)malononitrile, CCM: curcumin, *P. aeruginosa*: *Pseudomonas aeruginosa*, *S. aureus*: *Staphylococcus aureus*, NPs: CCM+TTD@ZIF‐8 NPs, UV‐vis: ultraviolet‐visible spectroscopy, ABDA: 9,10‐Anthracenediyl‐bis(methylene)dimalonic acid, ROS: reactive oxygen species, SEM： scanning electron microscopy.

The pH‐dependent release of CCM from CCM+TTD@ZIF‐8 NPs was identified by observing the CCM release behavior in various pH environments (pH 5.5, 6.5, and 7.5), mimicking pH changes in the microenvironment of an infected wound where pathogen growth could lead to glycolysis and acidification.^[^ ^24^ ^]^ A higher retention rate of CCM was observed at pH 7.5, whereas a burst release occurred at pH 5.5 and pH 6.5 over the initial 1 h, resulting in a ≈70% cumulative release ratio (Figure 2A). The greater release of CCM at pH 5.5 and pH 6.5 may be due to the acid‐responsive capacity of ZIF‐8, as confirmed by previous studies,^[^ ^4^ ^]^ with the disintegration of the dodecahedral structure in an acidic environment (Figure 2B), this is also evidenced by the release of Zn^2^^+^ under pH 5.5 conditions (Figure S4). Thus, the release of CCM could be accordingly adjusted by CCM+TTD@ZIF‐8 NPs through sensing pH change in the infected area.

The generation of ROS by CCM+TTD@ZIF‐8 NPs combined with blue light was identified using 9,10‐Anthracenediyl‐bis(methylene)dimalonic acid (ABDA) as the probe. ABDA, an anthracene derivative, shows four characteristic bands at 342, 359, 378 and 400 nm. It can specifically trap ^1^O_2_ by forming an endoperoxide product, leading to the disappearance of its characteristic absorption band. Thus, the reduction of its characteristic absorption peak indicates the consumption of ABDA and the corresponding ^1^O_2_ generation.^[^ ^25^ ^]^ When ABDA and CCM+TTD@ZIF‐8 NPs were solubilized with pH 7.4 PBS, no degradation of ABDA absorption was observed upon blue light irradiation (20 mW cm^−2^) at different times (0, 3, 5, 7, 9, 11, 13 and 15 min), indicating that blue light did not affect the absorption of ABDA (Figure 2C). However, in the presence of CCM+TTD@ZIF‐8 NPs, ABDA absorption decreased gradually with the increase in irradiation time (Figure 2D); an ≈20% decrease was observed after 15 min of blue light irradiation (Figure 2E), confirming the generation of ROS by CCM+TTD@ZIF‐8 NPs upon blue light irradiation. Meanwhile similar results were measured at pH 5.5, it can be shown that therapeutic photodynamic therapy can be performed under blue light irradiation either at the beginning when the wound pH is lowered or subsequently when the wound is alkaline (Figure S5). To verify the bacteria‐targeting and imaging properties of CCM+TTD@ZIF‐8 NPs, scanning electron microscopy (SEM) images and confocal fluorescence microscope images of bacteria following co‐incubation with CCM+TTD@ZIF‐8 NPs were obtained. SEM images showed that a large amount of *P. aeruginosa* and *S. aureus* stick to the surface of the CCM+TTD@ZIF‐8 NPs (Figure 2F), thus showing that CCM+TTD@ZIF‐8 NPs have excellent bacteria‐capturing abilities. Moreover, the CCM+TTD@ZIF‐8 NPs emitted red fluorescence under blue light irradiation and precisely merged with *P. aeruginosa* labelled with green fluorescence (Figure 2G), the same results were shown in animal experiments (Figure S6), in agreement with SEM observations. Thus, CCM+TTD@ZIF‐8 NPs have outstanding abilities for bacteria targeting and fluorescence imaging.

### 2.3 In vitro analysis of the bactericidal effect

The bactericidal effect of CCM+TTD@ZIF‐8 NPs combined with blue light irradiation was assessed using colony‐forming unit (CFU) assays. *S. aureus* (Gram‐positive) and *P. aeruginosa* (Gram‐negative) were chosen as models because they are the most common bacterial strains in infected wounds. The minimum inhibitory concentration (MIC) and minimum bactericidal concentration (MBC) of CCM@ZIF‐8 NPs and CCM+TTD@ZIF‐8 NPs, when combined with light irradiation against *S. aureus* and *P. aeruginosa*, were significantly lower than those of CCM@ZIF‐8 NPs and CCM+TTD@ZIF‐8 NPs alone. This indicated that the combination of CCM+TTD@ZIF‐8 NPs and blue light irradiation exerted excellent antibacterial activity. Thus, 62.5 µg mL^−1^ (2MIC) and 125 µg mL^−1^ (2MIC) of CCM+TTD@ZIF‐8 NPs were selected for subsequent bactericidal experiments against *S. aureus* and *P. aeruginosa*.

**FIGURE 3:**
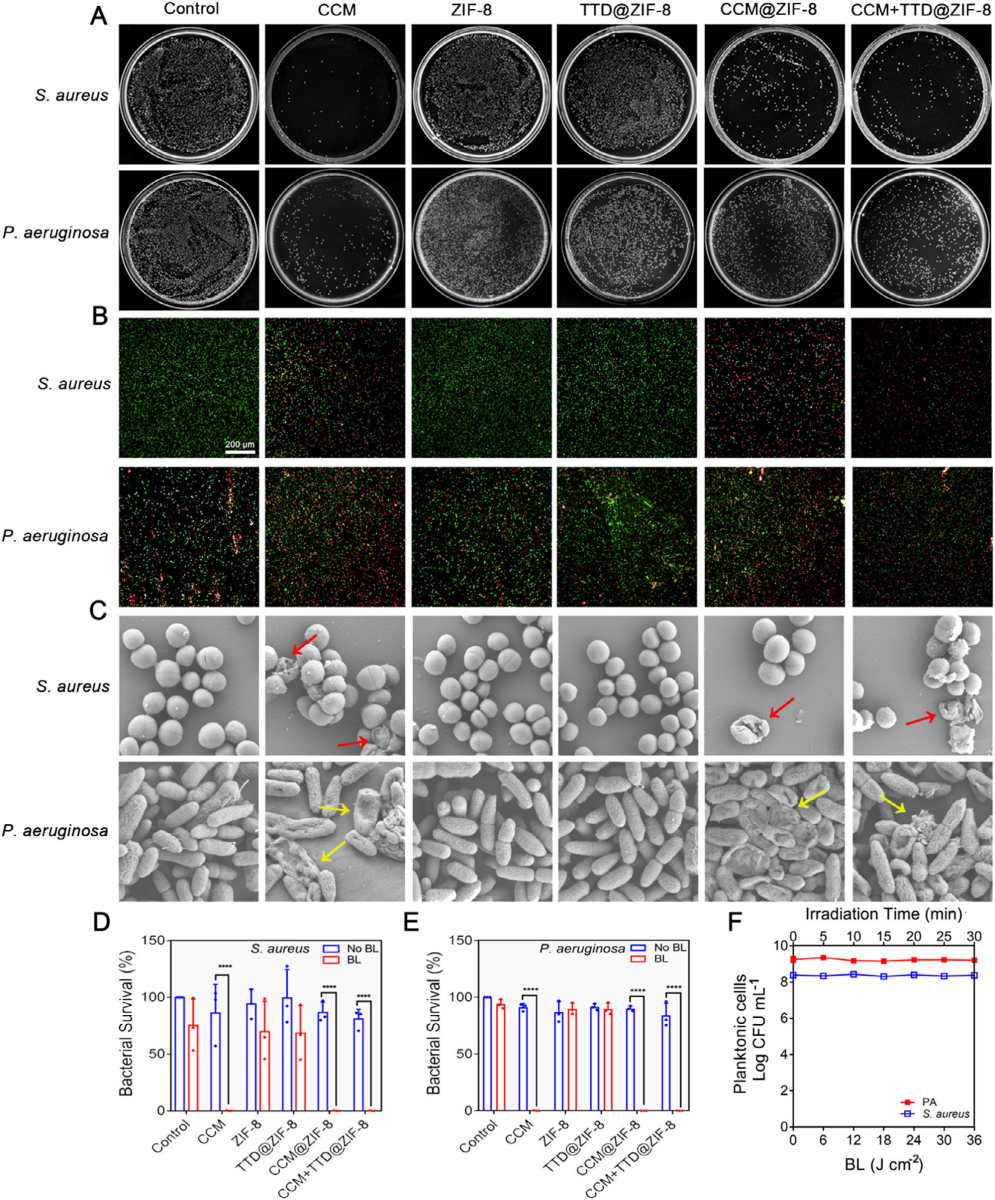
Bactericidal effect of CCM+TTD@ZIF‐8 NPs combined with blue light irradiation in vitro. (A) Colonies of *S. aureus* and *P. aeruginosa* on the LB plates. (B,C) Fluorescence microscopy images and SEM images of *S. aureus* and *P. aeruginosa*. (D,E) The bacterial survival rate of *S. aureus* and *P. aeruginosa*. (F) Colony count of different intensities of blue light (20 mW cm^−2^) against *S. aureus* and *P. aeruginosa*. Statistical analysis for bacterial survival rate was performed using paired *t*‐test. Data were displayed as mean ± SD (*n* = 3). *p ≤  0.05, **p ≤  0.01, ***p ≤  0.001. ZIF‐8: Zeolitic imidazolate framework 8, TTD: 2‐(2,6‐bis((E)−4‐(phenyl(4′‐(1,2,2‐triphenylvinyl)‐[1,1′‐biphenyl]−4‐yl)amino)styryl)−4H‐pyran‐4 ylidene)malononitrile, CCM: curcumin, *P. aeruginosa*: *Pseudomonas aeruginosa*, *S. aureus*: *Staphylococcus aureus*, CFU: colony‐forming unit, BL: blue light.

Prior to blue light irradiation, all groups showed the formation of large, viable bacterial colonies (Figure S7). Following irradiation, the CFU assay (Figure 3A) presented that CCM@ZIF‐8, and CCM+TTD@ZIF‐8 NPs exhibited noticeable bactericidal efficacy against *S. aureus* and *P. aeruginosa* in combination with blue light irradiation, which with CCM+TTD@ZIF‐8 NPs combined with blue light achieving an antibacterial rate of 99.84% against *S. aureus* and 99.94% against *P. aeruginosa*.

The results of Live/Dead staining images (Figure 3B and Figure S7) were consistent with the above data, with intense green fluorescence and negligible red fluorescence being observed in all groups without light irradiation as well as in the irradiated PBS, ZIF‐8, and TTD@ZIF‐8 groups. Following light irradiation combined with CCM, CCM@ZIF‐8, or CCM+TTD@ZIF‐8 NPs treatment, considerable red fluorescence was observed, suggesting that the membrane integrity of bacteria was damaged. *S. aureus* retained its spherical shape, and *P. aeruginosa* retained its rod shape following light irradiation combined with PBS, ZIF‐8, or TTD@ZIF‐8 treatment (Figure 3C). Collapsed membranes of *S. aureus* (red arrows) together with the deformed membranes of *P. aeruginosa* (yellow arrows) were observed following light irradiation combined with CCM, CCM@ZIF‐8, and CCM+TTD@ZIF‐8, in good agreement with the CFU assay (Figure 3D,E) and Live/Dead staining images.

Conversely, in the absence of NPs, bacterial strain viability was unaffected by irradiation with blue light at various intensities (0, 6, 12, 18, 24, 30, and 36 J cm^−2^) (Figure 3F), confirming that blue light did not affect bacterial activity. Minimal differences in MIC and MBC compared with treatment without light irradiation were observed following treatment with ZIF‐8 NPs + Light and TTD@ZIF‐8 NPs + Light (Figures S8 and S9), suggesting that TTD and ZIF‐8 alone did not induce bacterial death.

### 2.4 Biocompatibility evaluation

**FIGURE 4:**
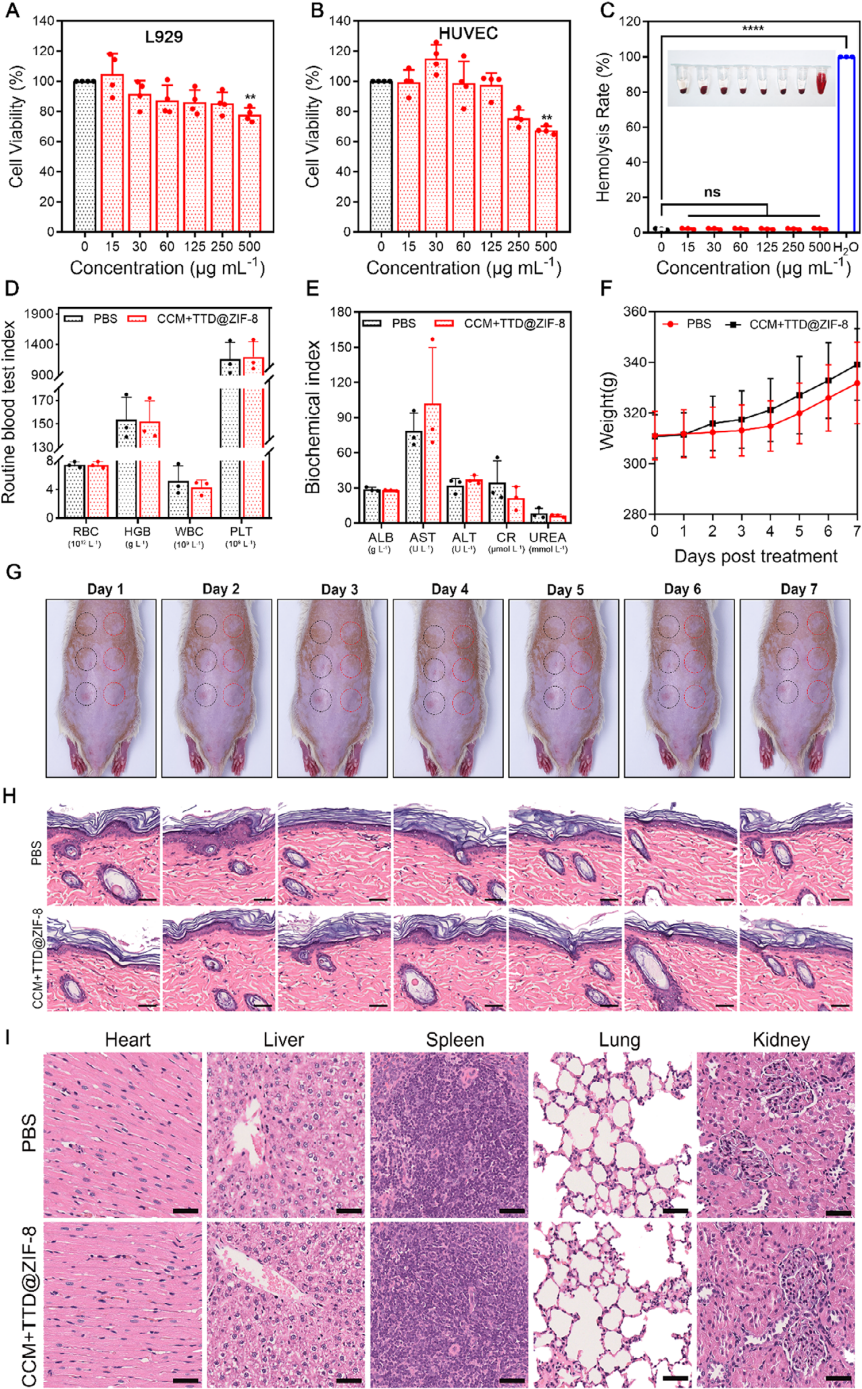
Biocompatibility evaluation of CCM+TTD@ZIF‐8 NPs. (A,B) CCK‐8 assay of cells at various concentrations (0−500 µg mL^−1^) of CCM+TTD@ZIF‐8 NPs (A: L929, B: HUVEC) (*n* = 4). (C) Hemolysis rate of RBC suspension following treatment with diverse concentrations (0−500 µg mL^−1^) of CCM+TTD@ZIF‐8 NPs (*n* = 3). (D,E) Routine blood test index and biochemistry index of SD rats after treatment of 125 µg mL^−1^ of the developed NPs or PBS for 7 days (*n* = 3). (F–H) Body weight, skin monitoring and histopathological assessment of healthy rats after treatment of 125 µg mL^−1^ of the developed NPs or PBS. Black circles represented PBS treatment and red circles represented CCM+TTD@ZIF‐8 NPs treatment (*n* = 3). Scale bars: 50 µm. (I) Histopathological assessment of major organs on day 7 after treatment of 125 µg mL^−1^ of the developed NPs or PBS. Scale bars: 50 µm. Statistical analysis for cell viability and hemolysis rate was performed using one‐way ANOVA with Tukey's post hoc test. Data were displayed as mean ± SD. *p ≤  0.05, **p ≤  0.01, ***p ≤  0.001. RBC: red blood cells, HGB: hemoglobin, WBC: white blood cells, PLT: platelets, ALB: albumin, AST: aspartate aminotransferase, ALT: alanine transaminase, CR: creatinine, L929: mouse fibroblast cells, L929 cells, HUVEC: human umbilical vein endothelial cells.

High concentrations of CCM and Zn^2+^ may exhibit cytotoxicity.^[^ ^26^ ^]^ Therefore, the in vitro and in vivo biocompatibility of CCM+TTD@ZIF‐8 NPs was tested. Figure 4A,B showed that CCM+TTD@ZIF‐8 NPs were biocompatible and suitable for applications, with high cell viability in L929 and HUVEC cells at concentrations not exceeding 250 µg mL^−1^ compared to untreated controls. The survival rate of the two types of cells began to show an obvious decrease at a concentration of 500 µg mL^−1^, likely due to the high concentration of CCM and Zn^2+^ released from CCM+TTD@ZIF‐8 NPs. The hemolysis assay for CCM+TTD@ZIF‐8 NP treatment at concentrations ranging from 0 to 500 µg mL^−1^ showed a colorless supernatant, similar to that in the PBS group, whereas that of the positive control (H_2_O) was red due to the lysis of RBCs (Figure 4C). As shown in Figure 4D,E, there were no significant abnormalities after treatment with CCM+TTD@ZIF‐8 NPs, suggesting that the developed NPs had no potential toxicity to blood cells and the hematopoietic system, and did not cause obvious dysfunction in the liver and kidney. This suggests that the morphology of RBCs in experimental groups remained intact. Additionally, following treatment with 125 µg mL^−1^ of CCM+TTD@ZIF‐8 NPs, a gradual increase in body weight was observed compared to rats in the PBS group (Figure 4F). There was no damage to the skin of rats after treating CCM+TTD@ZIF‐8 NPs compared with PBS treatment (Figure 4G,H). On day 7, the blood of rats was collected to test routine blood test index (red blood cells (RBC), hemoglobin (HGB), white blood cells (WBC), and platelets (PLT)) and biochemistry index (albumin (ALB), aspartate aminotransferase (AST), and alanine transaminase (ALT), and renal function‐related indicators, such as creatinine (CR) and urea. Histopathological results of various organs exhibited no pathological changes after treatment of CCM+TTD@ZIF‐8 NPs with 125 µg mL^−1^ for 7 days (Figure 4I). The above results demonstrated that CCM+TTD@ZIF‐8 NPs were biocompatible and suitable for applications.

The strong intensity of irradiated blue light could also lead to cytotoxicity due to the generation of cytotoxic ROS from intracellular chromophores (e.g., flavins and cytochromes).^[^ ^27^ ^]^ Thus, the effect of irradiation on L929 cells was also investigated. There was no significant difference in cell viability following irradiation at intensities of 6−18 J cm^−2^, but the cell viability was reduced following irradiation at 24−36 J cm^−2^ (Figure S10). The fluorescence intensity of live cells was comparable to that of the control group at a constant light density (20 mW cm^−2^) for 5−15 min, indicating the retention of the original morphology with high viability (Figures S11 and S12). Thus, blue light irradiation of 425 nm at 12 J cm^−2^ (20 mW cm^−2^ for 10 min) could be used in subsequent animal experiments.

### 2.5 In vivo treatment of infected burn wounds

**FIGURE 5:**
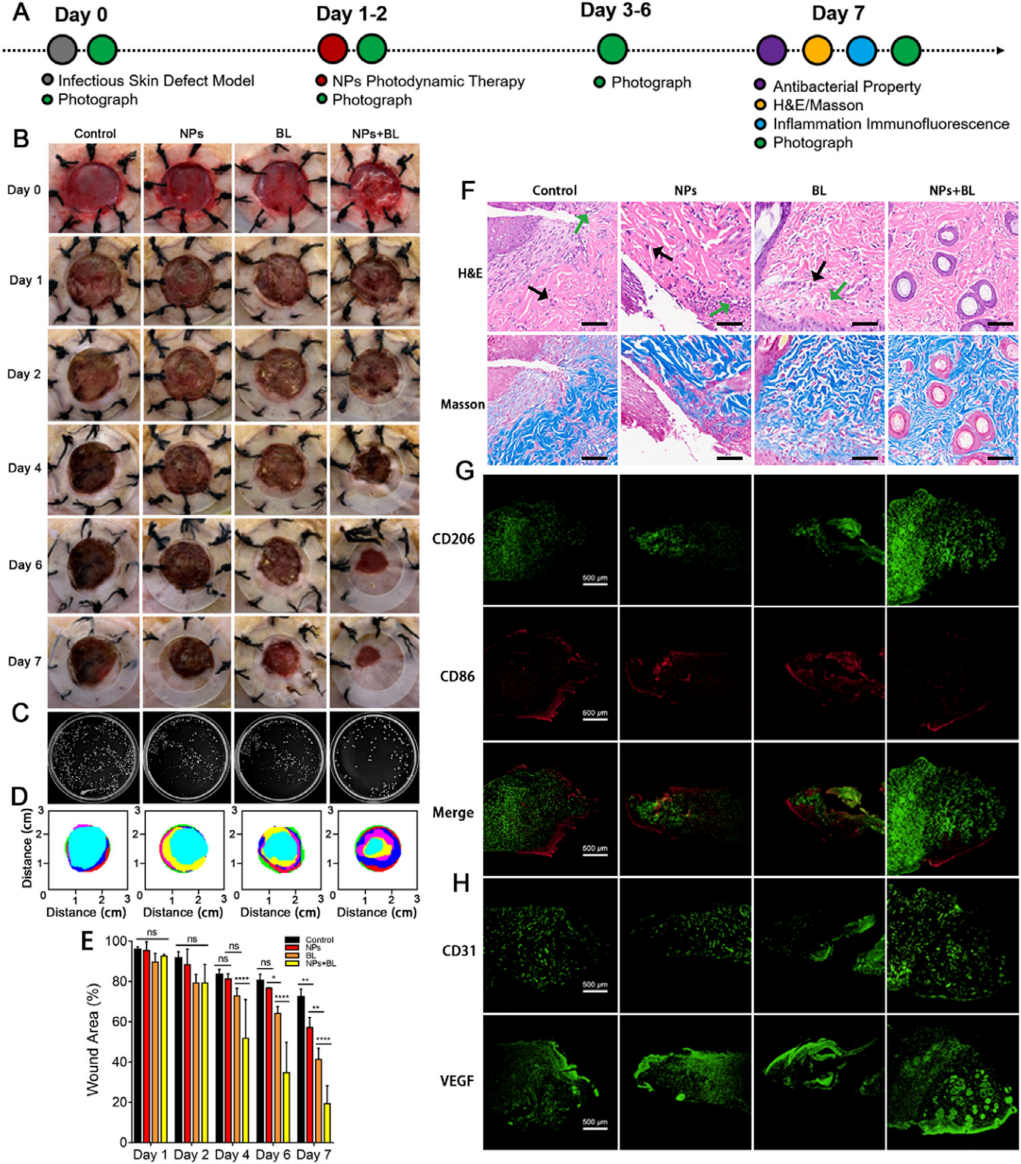
Treatment efficiency of CCM+TTD@ZIF‐8 NPs combined with blue light in infected burn wounds. (A) Experimental schedule of infected burn wounds in SD rats. (B) Corresponding photos of wounds in all groups over the study period (day 0, 1, 2, 4, 6, and 7). (C) Bacterial colonies of wounds in the four groups on day 7. (D) Schematic diagram of wound trace in four groups after treatment for 7 days. (E) Quantification of wound area (%) over time (*n* = 3). (F) H&E and Masson staining of skin tissue in all groups on day 7, bar = 100 µm. (G,H) Immunofluorescence images of CD206, CD86, CD31, and VEGF. Statistical analysis for wound area was performed using one‐way ANOVA with Tukey's post hoc test. Data were displayed as mean ± SD. *p ≤  0.05, **p ≤  0.01, ***p ≤  0.001. control: PBS, NPs: CCM+TTD@ZIF‐8 NPs, BL: blue light, VEGF: vascular endothelial growth factor.

To further demonstrate the therapeutic efficacy of CCM+TTD@ZIF‐8 NPs combined with blue light irradiation on infected burn wounds, an *S. aureus*‐infected subcutaneous wounded mouse model was established (Figure 5A) because *S. aureus* is the most common bacteria associated with infected wounds and shows high resistance to antibiotics.^[^ ^28^ ^]^ The efficiency of wound healing and wound bacterial load were monitored after treatment. Four groups were established in this experiment: the PBS (control) group, the pure CCM+TTD@ZIF‐8 NPs (NPs) group, the blue light (BL) group, and the CCM+TTD@ZIF‐8 NPs combined with blue light (NPs+BL) group. To minimize individual differences in rats, four infected burn wounds were established on the same rat. Treatments began one day after the establishment of the *S. aureus* infection, as observed by purulence (Figure 5B). The number of bacterial colonies on day 7 (Figure 5C) was recorded (Figure 5D) as was the wound trace of the different groups following treatment for 7 days. Compared to the control group, treatment with CCM+TTD@ZIF‐8 NPs plus blue light demonstrated have effective anti‐*S. aureus* properties, ascribed to the excellent PDT of CCM+TTD@ZIF‐8 NPs combined with blue light. Gradual wound scabbing and size reductions were observed for all groups to varying extents (Figure 5D,E). On day 4, the wound treated with CCM+TTD@ZIF‐8 NPs recovered slower than that treated with blue light alone and CCM+TTD@ZIF‐8 NPs plus blue light, showing an average wound area of 68.49%. However, wounds treated with blue light alone and CCM+TTD@ZIF‐8 NPs plus blue light showed early scab formation, with a rapid wound healing rate of 45.87% and 64.52%, respectively (Figure 5E). Thus, blue light significantly reduced wound area and led to early scab formation compared to PBS treatment, in agreement with previous reports.^[^ ^27^, ^29^ ^]^ The outstanding wound healing efficiency of blue light may be attributed to the increase in epithelialization and keratin‐10 mRNA levels upon blue light irradiation.^[^ ^27^ ^]^ Treatment with CCM+TTD@ZIF‐8 NPs plus blue light accelerated wound closure to the largest extent, with a wound area of 13.87% on day 7 (Figure 5E), suggesting that there was more coverage of new epithelial tissue under the synergistic efficiency of PDT and blue light. ROS levels were also detected in the wound tissue, demonstrating that curcumin can produce ROS under blue light irradiation, leading to a therapeutic effect (Figure S13). Furthermore, a negligible bactericidal effect was observed for either treatment alone (CCM+TTD@ZIF‐8 NPs or blue light), in agreement with the in vitro bactericidal effect.

The pathological situation of wounds was assessed using immunohistochemistry (H&E staining and Masson staining) and immunofluorescence (CD31 and VEGF staining, CD86 and CD206 staining) to evaluate the inflammation status, collagen deposition, and angiogenesis. H&E staining showed tissue necrosis accompanied by abundant inflammation‐related cell infiltration (black arrows) and cell vacuolization (green arrows) in samples treated with PBS, CCM+TTD@ZIF‐8 NPs, and blue light for 7 days (Figure 5F and Figure S14). In contrast, there were no inflammatory cells and cell vacuolization in response to combined CCM+TTD@ZIF‐8 NPs and blue light treatment, and the regeneration of hair follicles and epidermal tissue was also observed, demonstrating that the bactericidal effect of the CCM+TTD@ZIF‐8 NPs plus blue light accelerated epidermal recovery and wound healing. To further verify the inflammation state of the wound, immunofluorescence staining of CD86 and CD206 in all groups was performed. Compared to other groups, CCM+TTD@ZIF‐8 NPs plus blue light treatment led to a greater number of M2 macrophage cells and fewer M1‐type macrophages (Figure 5G and Figure S15), indicating that the photodynamic antibacterial effect of CCM+TTD@ZIF‐8 NPs plus blue light treatment contributed to the low numbers of bacteria on the wound, thereby alleviating the inflammatory response. Flow cytometry results were consistent with this (Figure S16). As a key component of the extracellular matrix, collagen is important for tissue remodeling.^[^ ^30^ ^]^ Herein, levels of collagen deposition were measured by Masson staining. Previous studies have reported that full‐thickness infected wounds need 14 days to acquire a high level of collagen deposition.^[^ ^31^ ^]^ Treatment with CCM+TTD@ZIF‐8 NPs plus blue light led to the formation of a larger area of collagen, with uniform and directional distribution, compared to other treatments on day 7, suggesting that CCM+TTD@ZIF‐8 NPs combined with blue light could accelerate collagen deposition. Insufficient collagen deposits in samples treated with PBS, CCM+TTD@ZIF‐8 NPs, or blue light may be attributed to the existence of bacteria. In addition, angiogenesis plays a major role in wound repair by facilitating the delivery of oxygen and nutrients to accelerate skin regeneration.^[^ ^32^ ^]^ CD31 and VEGF are considered markers of angiogenesis and the expression was monitored by immunofluorescence staining. There was minimal expression of CD31 and VEGF at the wound site following treatment with PBS, CCM+TTD@ZIF‐8 NPs, and blue light (Figure 5H and Figure S17), whereas clear expression in the combined CCM+TTD@ZIF‐8 NPs and blue light treatment group was observed, indicating that there was an effective result of angiogenesis following combined CCM+TTD@ZIF‐8 NPs and blue light treatment. Meanwhile, the inflammatory factors at the wound in the control group were significantly higher than those in the treatment group, which also indicated that combined CCM+TTD@ZIF‐8 NPs and blue light treatment could promote wound healing (Figure S17). Collectively, due to the excellent bactericidal performance of CCM+TTD@ZIF‐8 NPs combined with blue light, the recovery process of the infected wound was improved, as observed by a decrease in the inflammatory response, regeneration of hair follicles and epidermal tissue, and acceleration of collagen deposition and angiogenesis.

## 3 DISCUSSION

The bacteria targeted by ZIF‐8 is achieved through its positive charge on the surface. A good targeting effect was observed in the in vitro experiments, but in the process of in vivo application, we found that its specificity for targeting bacteria is not very effective. The resulting ROS released from CCM has the potential to damage normal tissue, but there was no specific targeted killing of drug‐resistant *S. aureus* and *P. aeruginosa* strains. Further research is needed to design targets that can specifically target bacteria in the in vivo environment, to improve the killing efficiency of drug‐resistant bacteria, improve the drug utilization of photosensitizer, and reduce the amount of drugs used in vivo to minimize toxic side effects. At the same time, the toxic side effects of ROS produced by photosensitizers on normal tissues are reduced, further improving the wound healing situation.

In the burn wound model, the in vivo imaging function seems to be used only as the localization of the material to explore its residence time at the wound and its co‐localization with bacteria, while its function of guiding therapy fails to exert its advantages in superficial trauma. We should focus on the specific killing of drug‐resistant bacteria and the acceleration of wound healing.

Based on the MOF material itself, it can have intrinsic biological activity through delivering specific metal ions or through catalysis.^[^ ^33^ ^]^ For example, the zinc ion in this study promotes cell growth and differentiation, nerve regeneration and broad‐spectrum antibacterial effect, making it an advantage in wound antibacterial and healing treatment. Furthermore, Cu‐BTC can catalyse the conversion of blood‐borne nitrosothiols to continuously release nitric oxide (NO).^[^ ^33b^ ^]^ NO has various functions, participating in biological processes such as inflammation, platelet adhesion, extracellular matrix (ECM) deposition, angiogenesis and healing.^[^ ^34^ ^]^ Working together with the copper ions with inherent properties, such as stimulating angiogenesis and increasing ECM deposition,^[^ ^35^ ^]^ is conducive to rapid wound healing.

Beyond that, it can also modify the composition of MOFs to exercise its inherent highly efficient antibacterial function and add additional biological effects by encapsulating drugs, ions, or unique bioactive agents. More importantly, we should develop the synergistic therapeutic potential of MOFs with various materials to produce even greater therapeutic effects, rather than simply mixing various components for in vivo treatment.

Finally, we should also emphasize personalized treatment, that is, to design unique and precise treatment plans for different tissue types, different pathological mechanisms of different diseases, and even the level of individual cell types, so that we have more in‐depth understanding and exploration of the relationship between the pathological mechanisms of diseases and the pharmacological effects of drugs, which is crucial for the development of materials, in‐depth drug studies, and the improvement in the treatment and recovery of human diseases.

## 4 CONCLUSIONS

Herein, for the first time, we constructed an intelligent and multifunctional theranostic system (CCM+TTD@ZIF‐8 NPs combined with blue light irradiation) for efficient bacterial capture and imaging along with pH response‐guided photodynamic sterilization to promote the healing of infected burn wounds. The positively charged surface of CCM+TTD@ZIF‐8 NPs enables accurate bacterial targeting at the infected sites, which not only promoted bacterial imaging but also triggered the maximized release of CCM and Zn^2+^ at the infected wound site. Thus, effective PDT was achieved upon blue light irradiation. Additionally, the efficient bactericidal and anti‐inflammatory properties of the system regulated the microenvironment in infected sites, thereby facilitating the regeneration of epidermal tissue and accelerating collagen deposition and angiogenesis. The results above demonstrate the powerful function of a multifunctional system in facilitating the effective healing of infected burn wounds. Therefore, the design of such a multifunctional system with bacteria capturing, imaging properties and efficient PDT provides a representative paradigm for the repair of infected burn wounds.

## 5 MATERIALS AND METHODS

### 5.1 Materials

Zinc nitrate hexahydrate (ZnNO_3_·6H_2_O, 99%) and methanol (CH_3_OH, 99.5%) were bought from the Chemical Reagent Factory (Guangzhou, China). 2‐Methylimidazole (2‐MIM; 98%) was bought from Macklin Biochemical Co., Ltd. (Shanghai, China). Curcumin (98%) was bought from Aladdin Biochemical Technology Co., Ltd. (Shanghai, China). 9,10‐Anthracenediyl‐bis(methylene)dimalonic acid (ABDA) and fuorescein isothiocyanate (FITC) were purchased from Sigma‐Aldrich Chemical. Co. (St. Louis, MO, USA). DAPI staining solution (KGA1808) was purchased from KeyGEN Biotechnology. Dimethyl sulfoxide (DMSO) and 4% paraformaldehyde (PFA) were obtained from Beijing Solarbio Science & Technology Co., Ltd. RNAase inhibitor (K1046) was obtained from APExBIO. Centrifuge tubes and glass‐bottom cell culture dishes were obtained from NEST Biotechnology. LIVE/DEAD Bac Light bacterial viability kit was bought from Thermo Fisher Scientific (Shanghai, China). Calcein/PI viability/cytotoxicity assay kit and cell proliferation kit (CCK‐8 assay) were bought from Beyotime Biotechnology (Shanghai, China). Mouse fibroblast cell line (L929) and human umbilical vein endothelial cell line (HUVEC) were purchased from the Shanghai Cell Bank of the Chinese Academy of Sciences. Rat TNF‐α, IL‐1β, IL‐6 and IFN‐γ ELISA kits were purchased from Jiangsu Meimian indusirial co., Ltd.

### 5.2 Sample preparation

TTD was synthesized according to a previous study by Liao et al.^[^ ^22a^ ^]^ The preparation of TTD‐loaded ZIF‐8, CCM‐loaded ZIF‐8, or ZIF‐8 loaded with both TTD and CCM was conducted based on a previously described method but with some modifications.^[^ ^36^ ^]^ Briefly, methanol (5 mL) containing ZnNO_3_·6H_2_O (150 mg) was added to methanol (10 mL) containing 2‐MIM (300 mg), TTD (1 mg) or CCM (10 mg), or both and sonicated for 2 h. The precipitate was collected via centrifugation (10,000 rpm, 15 min) and vacuum‐dried at 25°C for 24 h to obtain TTD@ZIF‐8 NPs, CCM@ZIF‐8 NPs, and CCM+TTD@ZIF‐8 NPs.

### 5.3 Sample characterization

X‐ray diffraction was performed on a Bruker D8 ADVANCE (Germany) diffractor equipped with a Cu Kα radiation source (*λ *= 1.5405 Å) in the 2*θ* range of 5°−40°. Attenuated total reflection Fourier transform infrared (ATR‐FTIR) spectrometry (IS50, USA) was conducted in the range from 4000 to 800 cm^−1^. The morphologies and elemental changes were characterized by scanning transmission electron microscopy (STEM; QUANTA 250 FEG, USA). Dynamic light scattering (DLS; Zetasizer Nano, UK) was employed to obtain the average size and polydispersity index of nanoparticles. UV–vis absorption spectra were recorded on a UV‐3600 instrument (Lambda 365, China). The CCM loading level in the NPs was determined from the CCM content in NPs compared to the added amounts. The OD425 nm value of CCM was recorded by microplate spectrophotometry (Synergy H1M, USA), and the CCM concentration was calculated based on the calibration curve. Fluorescence emission spectra were recorded using a fluorescence spectrophotometer (RF‐6000, Germany) at an excitation wavelength of 430 nm.

### 5.4 pH‐responsive behavior, photodynamic performance, bacterial capturing, and fluorescence imaging ability of CCM+TTD@ZIF‐8 NPs

The CCM release curve was monitored following the incubation of CCM+TTD@ZIF‐8 NPs in various pH buffers (pH 5.5, 6.5, and 7.5). At the predetermined timepoints (0, 20, 40, 60, 80, 100, 120, 140, and 180 min), 200 µL of the solution was withdrawn and the absorbance was detected at 425 nm. After 1 h of incubation, the precipitate was obtained through centrifugation and dried at 45°C for 24 h for scanning electron microscopy (SEM). ^1^O_2_ production was monitored using ABDA as the fluorescence probe. The mixtures of ABDA and CCM+TTD@ZIF‐8 NPs were exposed to 425 nm blue light and the UV–vis absorption spectrum was determined. To examine the bacterial capturing and fluorescence imaging properties, CCM+TTD@ZIF‐8 NPs were incubated with drug‐resistant bacteria (*S. aureus* and *P. aeruginosa*) for 30 min, fixed with pentanediol (2.5%) for 12 h, dehydrated using graded ethanol (30%, 50%, 70%, 80%, 90%, and 100%), and finally dried and imaged by SEM (SU8100, Japan). Green‐fluorescence labeled *P. aeruginosa* was incubated with the developed NPs for 30 min, and fluorescence imaging was observed by laser scanning confocal microscopy (LSCM; Nikon A1R, Japan).

### 5.5 Biocompatibility evaluation

For in vitro biosafety assessment, the developed NPs (CCM+TTD@ZIF‐8 NPs) with different concentrations (0, 15, 30, 60, 125, 250, and 500 µg mL^−1^) were incubated with two kinds of cells (L929, HUVEC) for 24 h. L929 and HUVEC were used as models due to their vital role in wound healing. Subsequently, CCK‐8 dye was added to the mixtures and cultivated for 1.5−2 h in the dark and OD_450_ values were read using a microplate reader (BIO‐EAD, USA). The hemolysis assay was also used to determine the toxicity of NPs. Fresh sheep blood was used to test the hemolysis of the developed NPs at different concentrations (0, 15, 30, 60, 125, 250, and 500 µg mL^−1^). The experiment was approved by the Experimental Animal Ethics Committee of Southern Medical University, Guangzhou, China. Fresh sheep blood was obtained from Guangzhou Hongquan Biological Technology Co., Ltd., China. Briefly, sheep blood was centrifuged at 1500 rpm min^−1^ for 30 min and washed with PBS several times until the supernatant was clear. The samples (1 mL) were incubated with the above precipitation (red blood cells; RBCs) at room temperature for 2 h. PBS and pure water were set as negative (hemolysis rate = 0%) and positive (hemolysis rate = 100%) controls, respectively. The OD_545nm_ value of the supernatants was obtained using the microplate reader to calculate the rate of hemolysis as follows:

$$\mathrm{Hemolysis}\;\mathrm{rate}\;\left(\%\right)=\frac{A_{\mathrm{Sample}}-A_{\mathrm{Negative}}}{A_{\mathrm{Positive}}-A_{\mathrm{Negative}}}$$

The toxicity of different intensities of blue light (0, 6, 12, 18, 24, 30, and 36 J cm^−2^) was evaluated by CCK‐8 and Live/Dead assays. For Live/Dead assays, a working concentration of 3 µm was incubated directly with these treated cells for 20 min in the dark, washed several times with phosphate‐buffered saline (PBS), and imaged using an inverted fluorescence microscope (ECLIPSE Ti2‐U, Japan). The Live/Dead fluorescence images were analyzed using ImageJ software.

For in vivo biocompatibility evaluation of CCM+TTD@ZIF‐8 NPs, healthy male SD rats (301−350 g body weight, three for each group) were anesthetized (isoflurane) and depilated, PBS and CCM+TTD@ZIF‐8 NPs (125 µg mL^−1^) were added to the depilated back of rats. The body weights of rats were monitored for 7 days after treatment. At 7 days, blood was collected to test the routine blood test indices and biochemical index of mice. Additionally, vital organs including the heart, liver, spleen, lung, and kidney of rats were harvested for histological analysis.

### 5.6 In vitro antibacterial property of CCM+TTD@ZIF‐8 NPs combined with blue light

Drug‐resistant *S. aureus* and *P. aeruginosa* were chosen as representative bacteria to test the antibacterial activities of developed NPs combined with blue light via a colony‐counting method, Live/Dead staining, and SEM. Prior to the experiments, the minimum inhibitory concentration (MIC) and minimum bactericidal concentration (MBC) of the developed NPs were confirmed by the double dilution method at concentrations ranging from 62.5 to 500 µg mL^−1^. Subsequently, diluted bacterial suspensions (1 × 10^8^ CFU mL^−1^) were incubated with the developed NPs dispersion (the concentration of 2MIC) for 3 h with or without blue light irradiation (20 mW cm^−2^, 10 min). Finally, the treated suspension was diluted 10^4^ times using PBS and spread on a solid LB agar plate at 37°C for 18 h. All experiments were performed in triplicate.

For Live/Dead assays, a working concentration of 3 µm was prepared according to the manufacturer's protocol, added to the treated suspensions, and incubated for 15−20 min in the dark. The stained cells were then imaged using an inverted fluorescence microscope (ECLIPSE Ti2‐U, Japan).

For SEM observation, the treated mixtures of bacterial cells and NPs were centrifuged and washed f times with PBS. The bacterial cells were fixed with glutaraldehyde (2.5%) for 12 h, then dehydrated in graded alcohol (30%, 50%, 70%, 80%, 90%, 100%) for 5 min, and freeze‐dried for SEM imaging.

### 5.7 Wound healing assays

In vivo, the evaluation of wound healing following treatment with CCM+TTD@ZIF‐8 NPs combined with blue light was achieved by constructing infected burn wounds on the skin of SD rats. The animal experimental protocol was reviewed by the Experimental Animal Ethics Committee of Guangdong Provincial Medical Laboratory Animal Center and was in compliance with the relevant ethical regulations (approval number: C20230910). Male rats (301−350 g body weight) were obtained from Guangzhou Ruige Biological Technology Co., Ltd., China. All animals were housed in individual cages under constant temperature (22°C) and humidity with a 12‐h light and a 12‐h dark cycle, and they had access to food and water ad libitum throughout the study. The experiments were repeated twice or three times. Rats were anesthetized (isoflurane) and the dorsal hair of the rats was removed, and then the burn instrument was preheated for 5 min up to 95°C and placed on the dorsal side of the rat for 10 s, and the burned skins were excised and a burn wound of 12 mm was then established on their backs. The other three burns were made as symmetrically as possible on the back, resulting in four full‐thickness burns. In order to avoid variations in the creation of the burns, one person created all the burns.^[^ ^37^ ^]^ The burn wounds were then inoculated with drug‐resistant *S. aureus* (20 µL, 1.0 × 10^8^ CFU mL^−1^) and secured with a rubber ring. The defects were cured after 24 h. The rats were randomly divided into four groups (control, CCM+TTD@ZIF‐8 NPs (NPs) group, blue light (BL) group, and CCM+TTD@ZIF‐8 NPs combined with blue light (NPs+BL) group) with four mice in each group. The wound area was photographed on specific days (0, 1, 2, 4, 6, and 7 days) and quantified with Image J software. Skin tissues on day 7 were then excised from the wounds and fixed with the 4% paraformaldehyde solution for pathological evaluation. Hematoxylin and eosin (H&E) staining and Masson staining were conducted to assess the pathological conditions of the wounds. Immunofluorescence staining for CD86 and CD206 was used to display the balance of M1 and M2 macrophages, thereby evaluating the wound inflammation status. Angiogenesis was assessed by immunofluorescence staining for CD31 and VEGF. The bacteria numbers from the excised wound tissue were counted using the flat colony‐counting method. Briefly, the excised wound tissue was resuspended in 1 mL of PBS, and the homogenates obtained after vortex and sonication were serially diluted in PBS and spread on a solid LB agar plate at 37°C for 24 h.

### 5.8 Statistical analysis

All experiments were performed at least three times, and all data were presented as the mean ± standard deviation (SD). Statistical differences were analyzed by one‐way analysis of variance (ANOVA) (*p < 0.05, **p < 0.01, and ***p < 0.001). GraphPad Prism 8.0 was used for statistical analysis, and Image J software was used to analyze images of the fluorescence and wound area.

## CONFLICT OF INTEREST STATEMENT

The authors declare no conflicts of interests.

## Supporting information

Supporting Information

## Data Availability

The raw data and processed data required to reproduce these findings are available from the corresponding author upon request.
