# Correction to "Intelligent Bacteria‐Targeting ZIF‐8 Composite for Fluorescence Imaging‐Guided Photodynamic Therapy of Drug‐Resistant Superbug Infections and Burn Wound Healing"

**DOI:** 10.1002/EXP.20250443

**Published:** 2025-07-17

X. Li, W. Wang, Q. Gao, et al., “Intelligent Bacteria‐Targeting ZIF‐8 Composite for Fluorescence Imaging‐Guided Photodynamic Therapy of Drug‐Resistant Superbug Infections and Burn Wound Healing,” *Exploration (Beijing, China)* 4, no. 6 (2024): 20230113. https://doi.org/10.1002/EXP.20230113.

The images in Figure 4G (skin photographs) and Figure 5C (bacterial plate—NPs group) were erroneously utilized. The authors identified these inaccuracies and have provided the corrected versions of Figures 4G and 5C below:

**FIGURE 4G:**
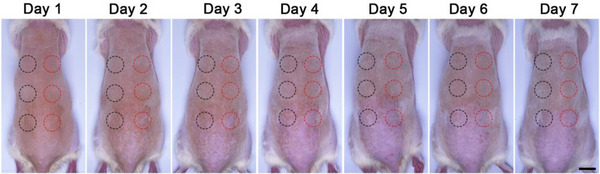
Skin monitoring of healthy rats after treatment of 125 µg mL^−1^ of the developed NPs or PBS. Black circles represented PBS treatment and red circles represented CCM+TTD@ZIF‐8 NPs treatment (*n* = 3). Scale bars: 2 cm.

**FIGURE 5C:**
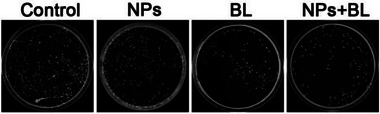
Bacterial colonies of wounds in the four groups on day 7. Statistical analysis for wound area was performed using one‐way ANOVA with Tukey's post hoc test. Data were displayed as mean ± SD. *p≤ 0.05, **p≤ 0.01, ***p≤ 0.001.

We apologize for this error.

