# Supplementary material for: Intelligent bacteria‐targeting ZIF‐8 composite for fluorescence imaging‐guided photodynamic therapy of drug‐resistant superbug infections and burn wound healing
Source: Exploration (Beijing). 2024 Apr 19;4(6):20230113. doi: 10.1002/EXP.20230113 (PMC11655311; doi:10.1002/EXP.20230113)
Supplement: Supplementary file 1 — Supporting Information [file EXP2-4-20230113-s001.docx]

**Intelligent bacteria-targeting ZIF-8 composite for fluorescence imaging-guided photodynamic therapy of drug-resistant superbug infections and burn wound healing**

Xiaoxue Li ^a, 1^, Wei Wang ^a, 1^, Qiuxia Gao ^a, b, 1^, Shanshan Lai ^a, 1^, Yan Liu ^c^, Sitong Zhou ^d^, Yan Yan ^a^, Jie Zhang ^a^, Huanhuan Wang ^a^, Jiamei Wang ^a^, Yi Feng ^a^, Ronghua Yang ^e, *^, Jianyu Su ^f, *^, Bin Li ^b, *^, Yuhui Liao ^a, *^

^a^ Molecular Diagnosis and Treatment Center for Infectious Diseases, Dermatology Hospital of Southern Medical University, Guangzhou 510091, Guangdong, China

^b^ School of Inspection, Ningxia Medical University, Yinchuan 750004, Ningxia, China

^c^ Institute for Health Innovation and Technology, National University of Singapore, Singapore 117599, Singapore

^d^ Department of Burn Surgery & Department of Dermatology, The First People’s Hospital of Foshan, Foshan 528000, Guangdong, China

^e^ Department of Burn and Plastic Surgery, Guangzhou First People's Hospital, South China University of Technology, Guangzhou 510030, Guangdong, China

^f^ School of Food Science and Engineering, South China University of Technology, Guangzhou 510640, Guangdong, China

* Corresponding authors:

Prof. R. H. Yang

Department of Burn and Plastic Surgery, Guangzhou First People's Hospital, South China University of Technology, Guangzhou 510030, Guangdong, China

E-mail: 21720091@qq.com

Prof. J. Y. Su

School of Food Science and Engineering, South China University of Technology, Guangzhou 510640, Guangdong, China

E-mail: [jysu@scut.edu.cn](mailto:jysu@scut.edu.cn)

Dr. Bin Li

School of Inspection, Ningxia Medical University, Yinchuan 750004, Ningxia, China

E-mail: [libin63@mail2.sysu.edu.cn](mailto:libin63@mail2.sysu.edu.cn)

Prof. Y. H. Liao

Molecular Diagnosis and Treatment Center for Infectious Diseases, Dermatology Hospital of Southern Medical University, Guangzhou 510091, Guangdong, China

E-mail: liaoyh8@mail.sysu.edu.cn

^1^ Co-first author: These authors contributed equally to this work.


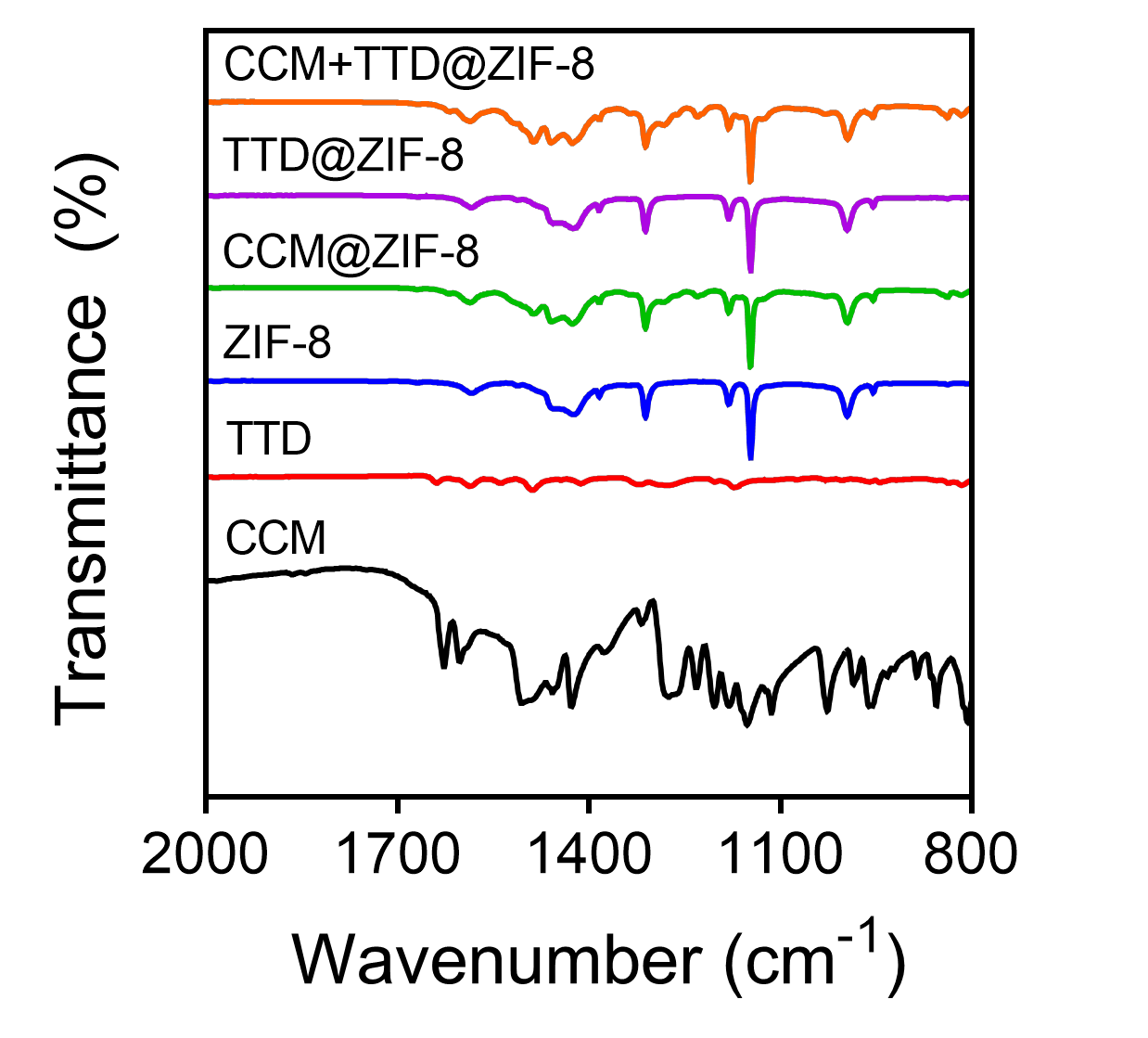


**Figure S1.** FTIR spectra of CCM, TTD, ZIF-8, CCM@ZIF-8, TTD@ZIF-8 and CCM+TTD@ZIF-8.

**
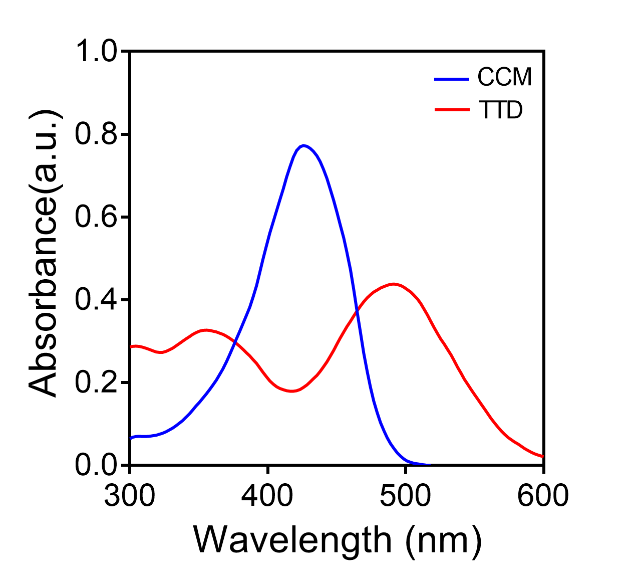
**

**Figure S2.** UV-Vis absorption spectra of CCM and TTD.

**
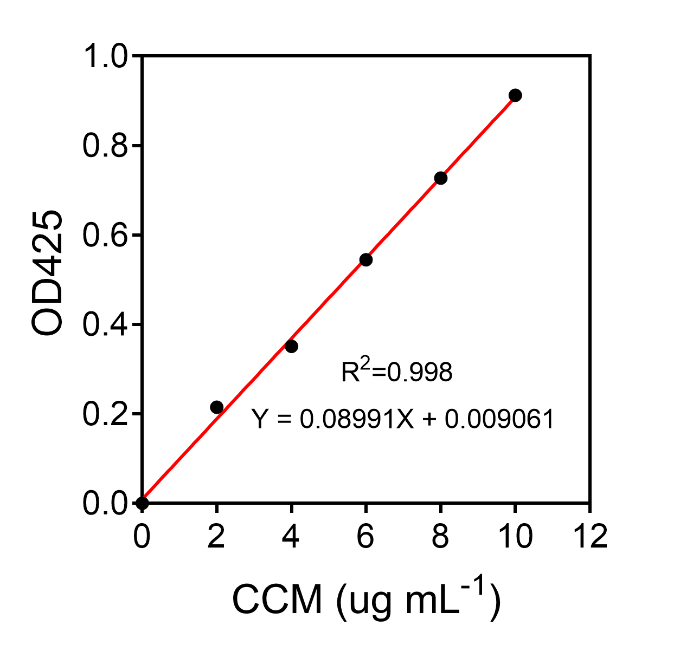
**

**Figure S3.** Concentration–absorbance standard curve of CCM. Equation of the standard curve is Y=0.08991X + 0.009061 (R^2^=0.998).


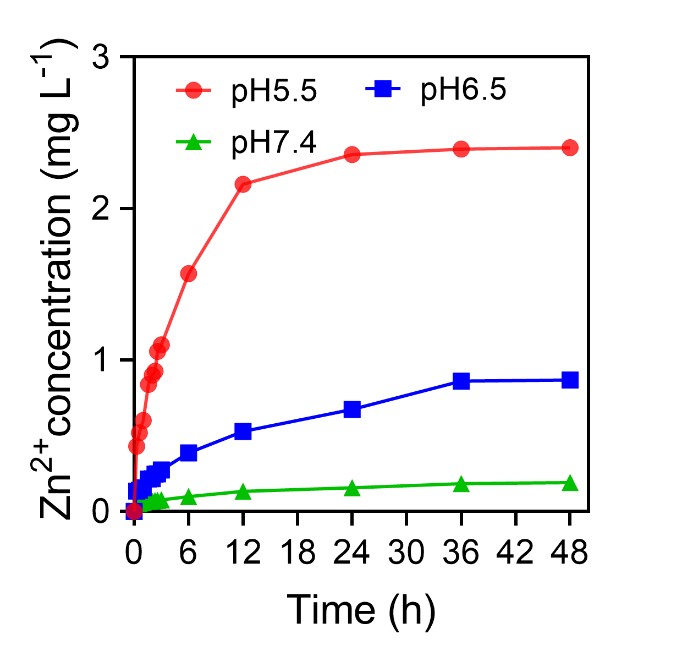


**Figure S4.** Zn^2+^ release profile from CCM+TTD@ZIF-8 NPs in PBS at pH 5.5, 6.5, and 7.5.


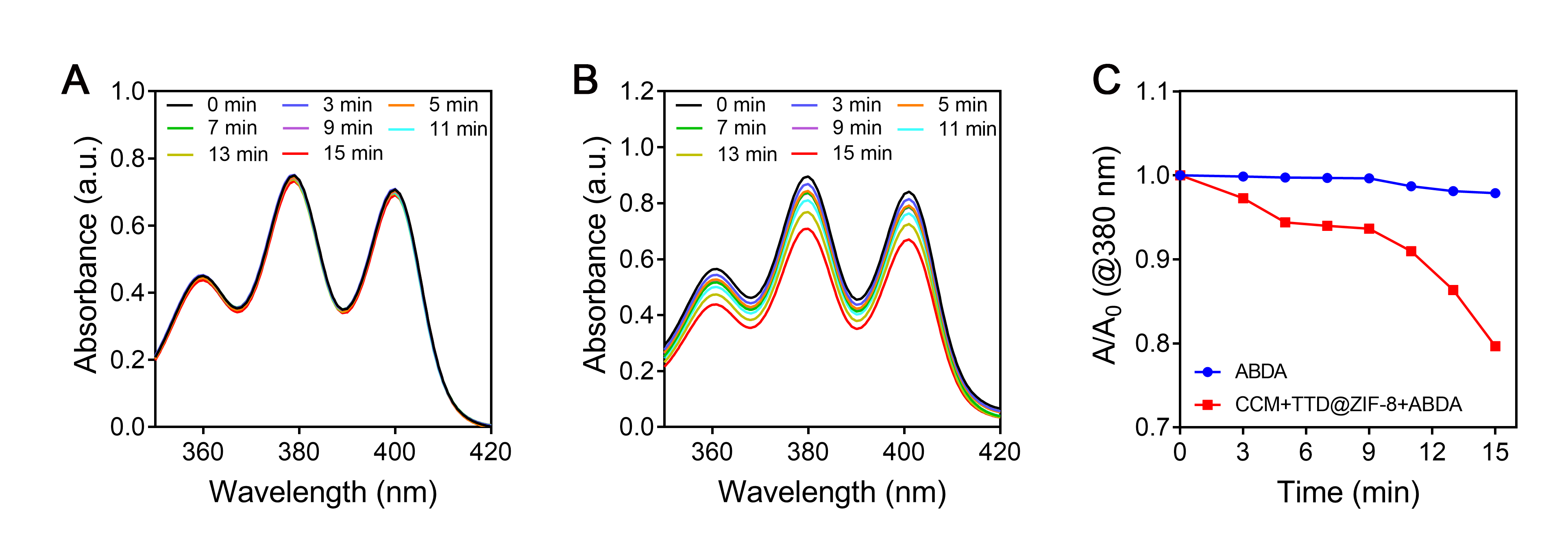


**Figure S5.** (A) UV-vis absorption spectra of ABDA under blue light irradiation (20 mW cm^-2^) with different times (0, 3, 5, 7, 9, 11, 13, and 15 min). (B) UV-vis spectra of ABDA in the presence of CCM+TTD@ZIF-8 NPs under blue light irradiation (20 mW cm^-2^) at different times (0, 3, 5, 7, 9, 11, 13, and 15 min). (C) Comparison of ROS generation ability between ABDA and ABDA+NPs under blue light irradiation. ABDA was solubilized with CCM+TTTD@ZIF-8 NPs in PBS at pH 5.5.


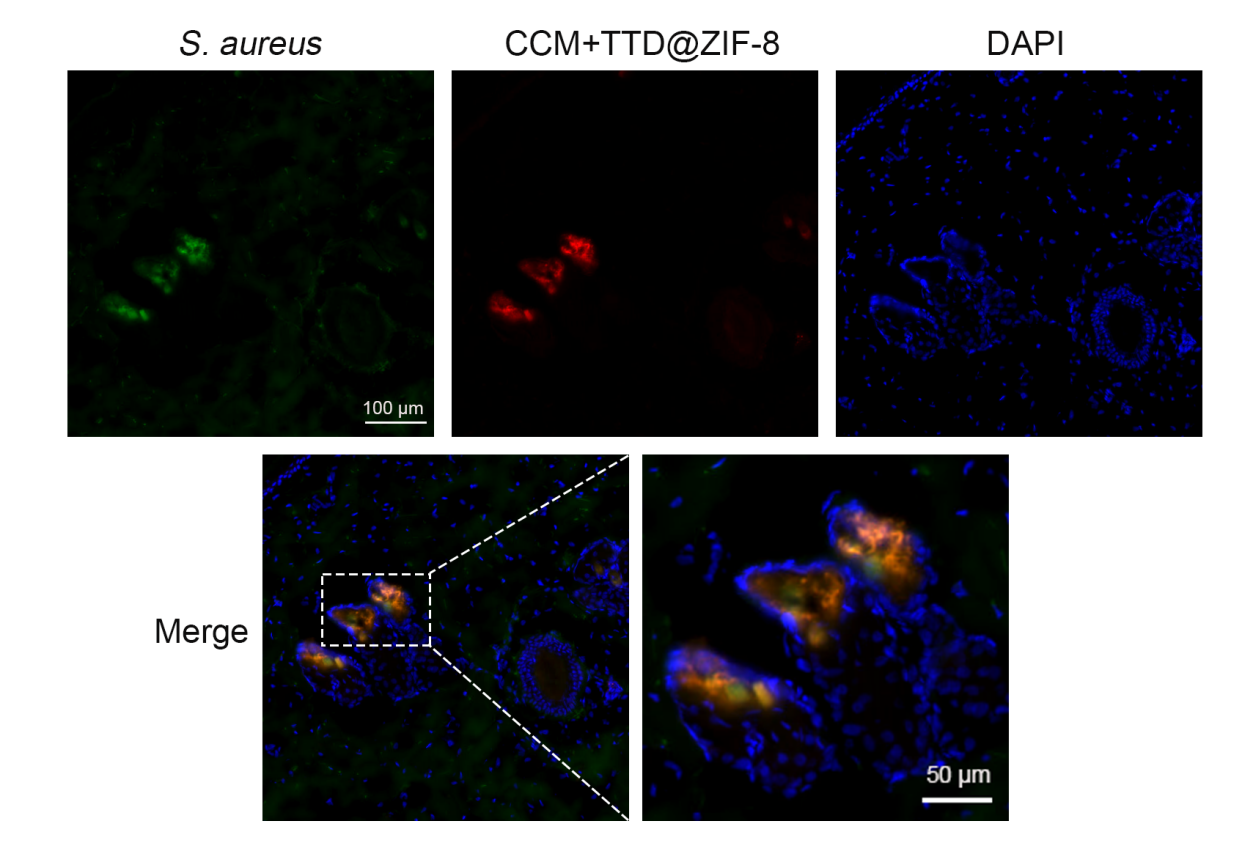


**Figure S6.** Fluorescence images of the infected skin slice of rat with CCM+TTD@ZIF-8 (red) and *S. aureus* -FITC (green). The nuclei was stained with DAPI (blue). The image on the right indicates the enlarged view of the box shown on the left image.

**
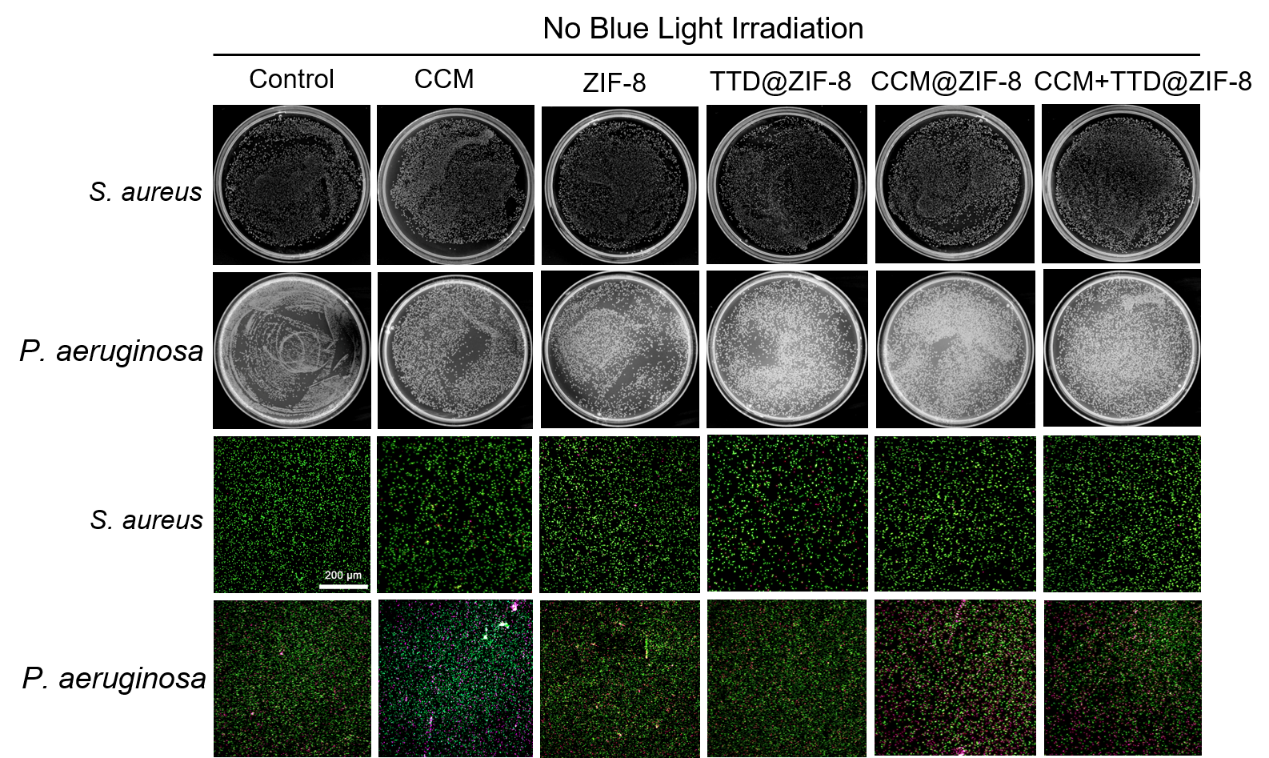
**

**Figure S7.** Colonies and live/dead staining images of *S. aureus* and *P. aeruginosa* without blue light irradiation.


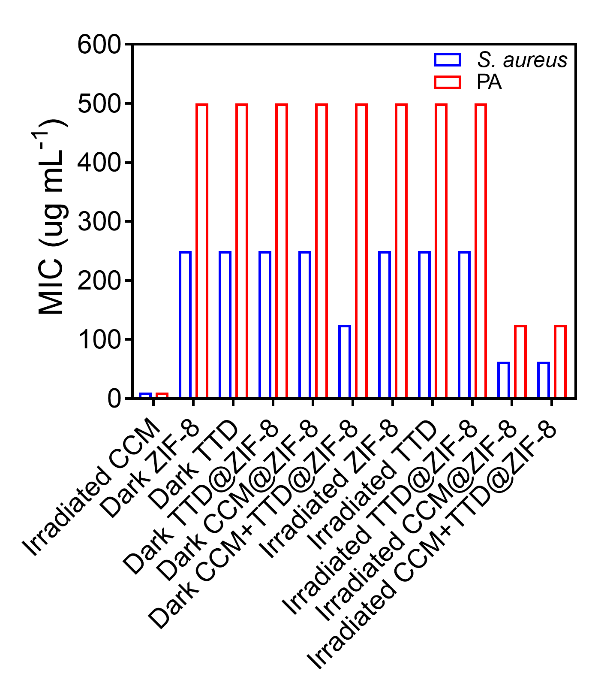


**Figure S8.** Minimum inhibitory concentration of CCM, ZIF-8, TTD, TTD@ZIF-8, CCM@ZIF-8, CCM+TTD@ZIF-8 nanoparticles with or without blue light irradiation.


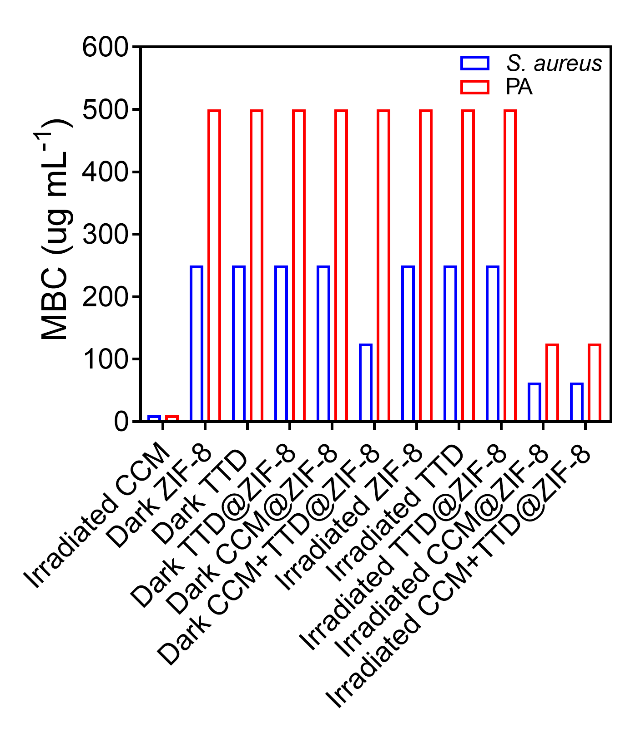


**Figure S9.** Minimum bactericidal concentration of CCM, ZIF-8, TTD, TTD@ZIF-8, CCM@ZIF-8, and CCM+TTD@ZIF-8 nanoparticles with or without blue light irradiation.


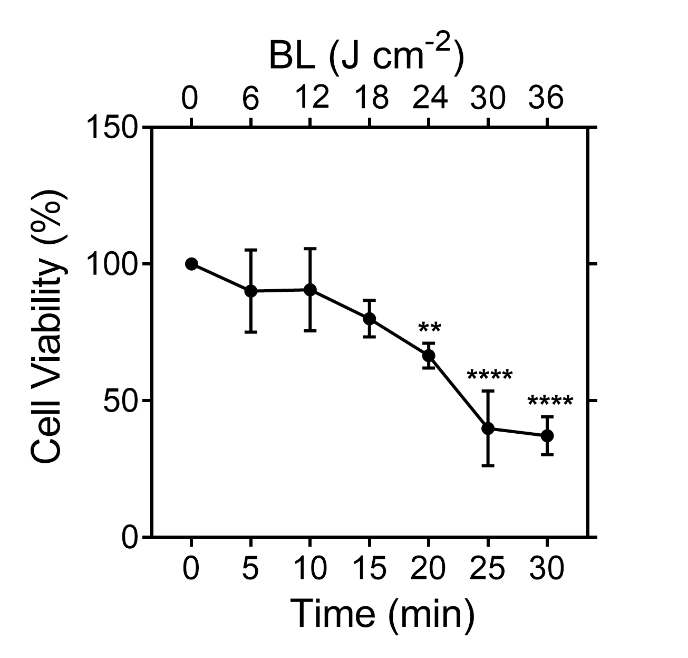


**Figure S10.** CCK-8 assay of L929 cells under blue light irradiation at different intensities (0–36 J cm^-2^). Statistical analysis for cell viability was performed using one-way ANOVA with Tukey’s post hoc test. Data were displayed as mean ± SD (n=3). *P≤ 0.05, **P≤ 0.01, ***P≤ 0.001.

**
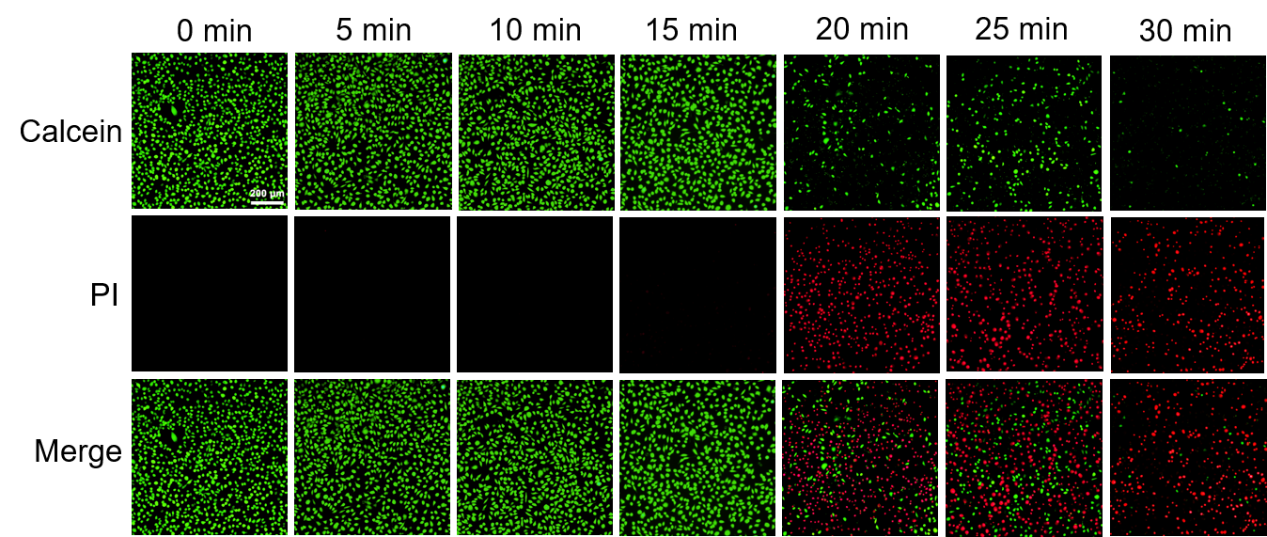
**

**Figure S11.** Live/dead staining images of L929 cells under blue light irradiation at different intensities.

**
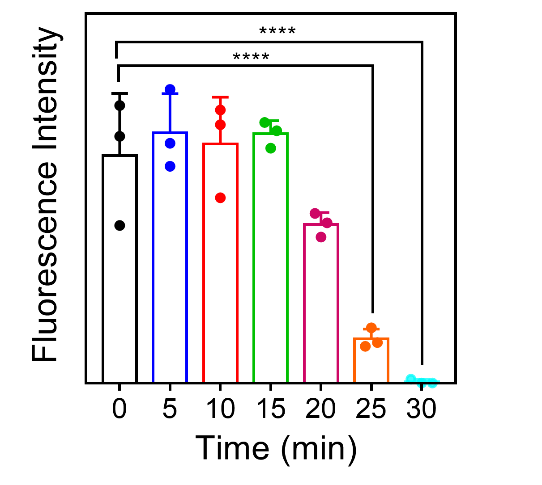
**

**Figure S12.** Quantification of the fluorescence intensity of L929 cells. Statistical analysis for fluorescence intensity was performed using one-way ANOVA with Tukey’s post hoc test. Data were displayed as mean ± SD (n=3). *P≤ 0.05, **P≤ 0.01, ***P≤ 0.001.


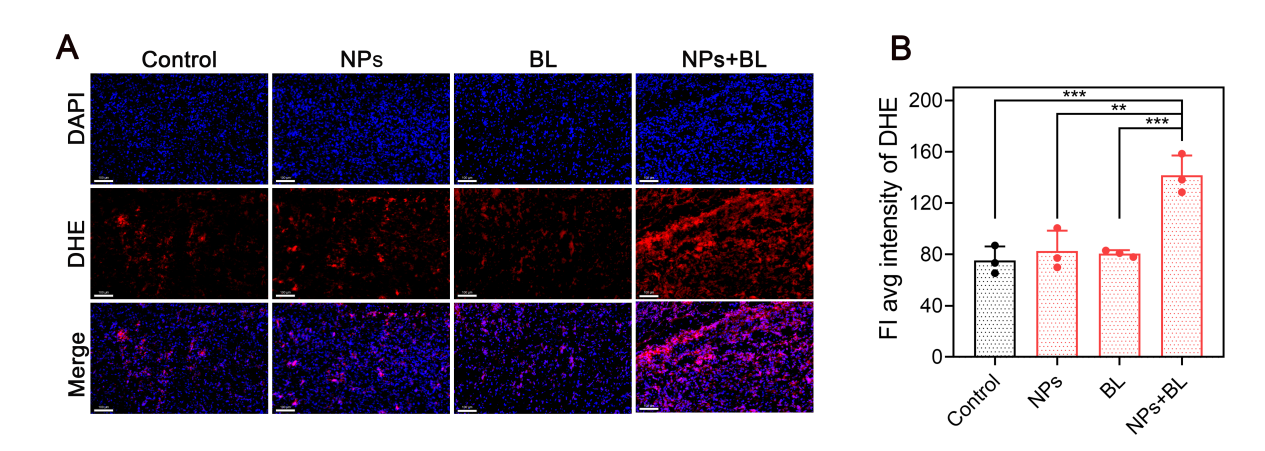


**Figure S13.** (A) DHE staining of wound after different treatments (red: DHE; blue: DAPI). (B) Relative DHE fluorescence intensity in different groups. Data are mean±SD, n = 3. *P≤ 0.05, **P≤ 0.01, ***P≤ 0.001.


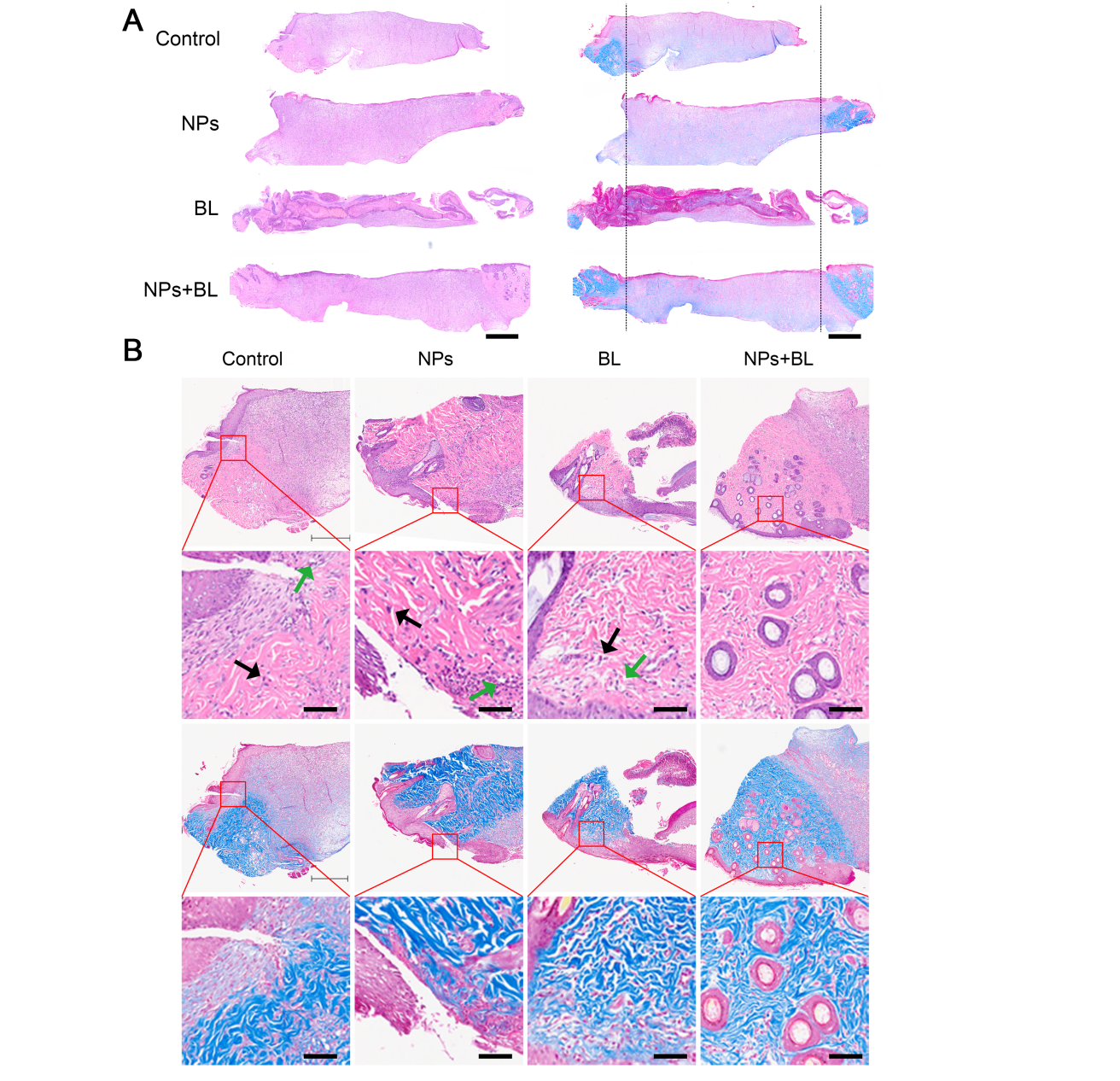


**Figure S14.** H&E and Masson staining of skin tissue in all groups on day 7, bar = 1 mm (A). bar = 100 μm (B).

**
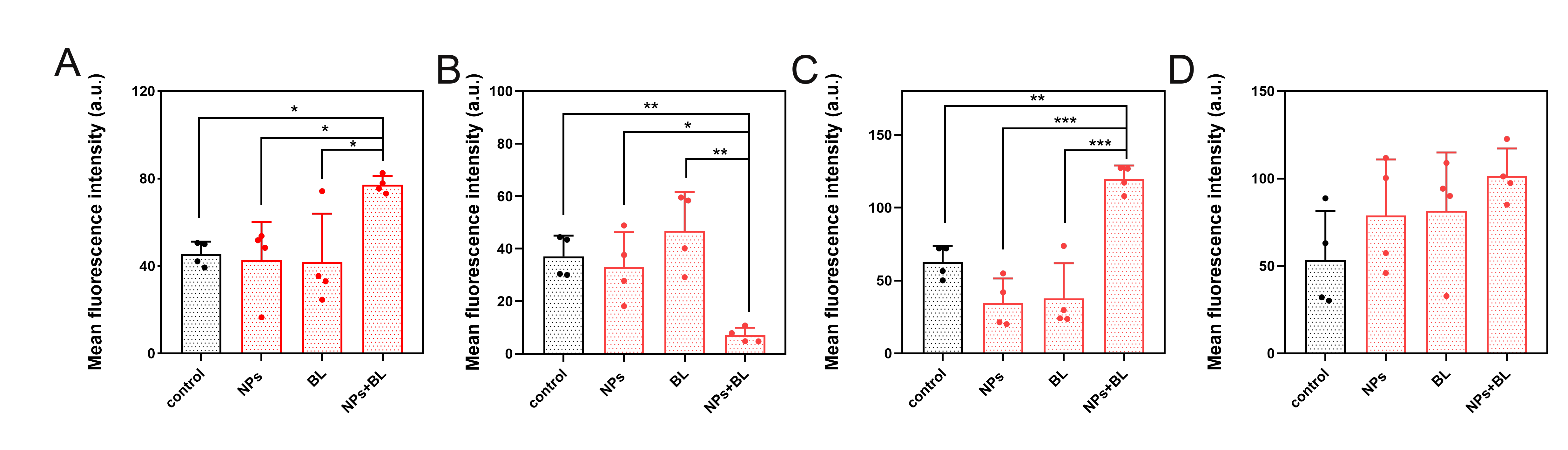
**

**Figure S15**. Fluorescence intensity analysis of CD31 (A), CD 86 (B), CD206 (C) and VEGF (D). Statistical analysis for fluorescence intensity was performed using one-way ANOVA with Tukey’s post hoc test. Data were displayed as mean ± SD (n = 4). *P≤ 0.05, **P≤ 0.01, ***P≤ 0.001.


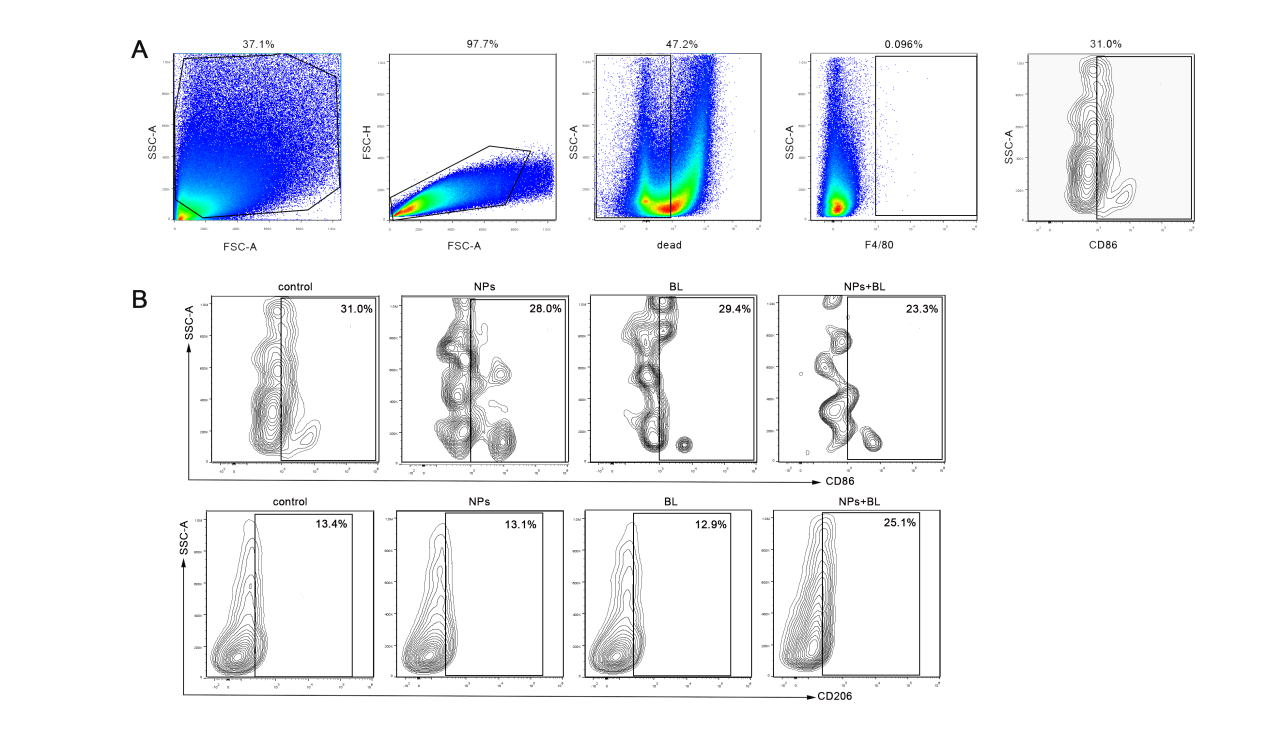


**Figure S16.** Representative flow cytometry plots detecting the ratio of M1 (A) and M2 (B) macrophages in wound tissues after different treatments.


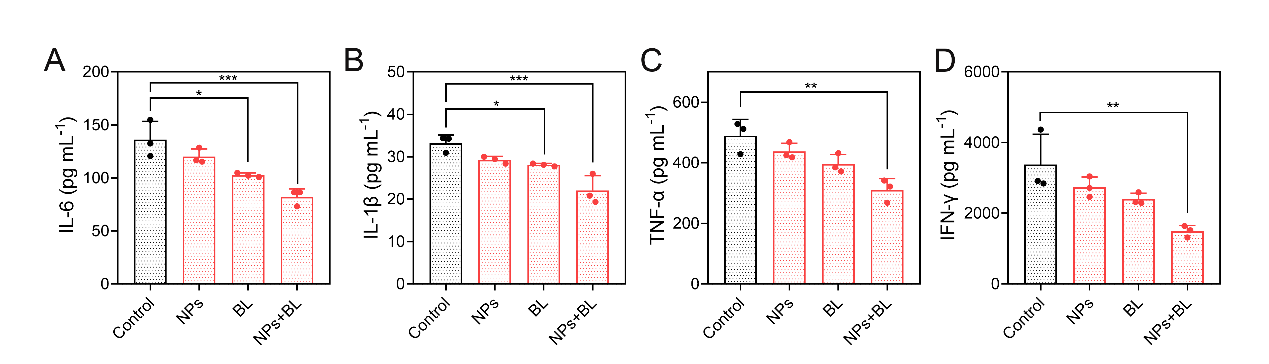


**Figure S17**. ELISA detection of varied pro-inflammatory cytokines in the healing of wounds 7 days after different treatments. Data were displayed as mean ± SD (n = 3). Statistical significance was calculated *via* ordinary one-way ANOVA. P**<0.01, P***<0.001.

**Table S1.** Drug-loading capacity (DLC) and drug-loading encapsulation (DLE) of CCM@ZIF-8 nanoparticles and CCM+TTD@ZIF-8 nanoparticles.

|  | CCM@ZIF-8 | CCM+TTD@ZIF-8 |
| --- | --- | --- |
| DLC (%) | 4.77% | 5.12% |
| DLE (%) | 19.07% | 20.46% |
